# Two novel viruses associated with the *Apis mellifera* pathogenic mite *Varroa destructor*

**DOI:** 10.1038/srep37710

**Published:** 2016-11-24

**Authors:** Sofia Levin, Noa Sela, Nor Chejanovsky

**Affiliations:** 1 Department of Entomology, Institute of Plant Protection, Agricultural Research Organization, The Volcani Center, Rishon LeZion, 7528809, Israel; 2 Institute of Agroecology and Plant Health, Robert H. Smith Faculty of Agriculture, Food and Environment, Hebrew University of Jerusalem, Rehovot, 76100, Israel; 3 Department of Plant Pathology and Weed Research, Institute of Plant Protection, Agricultural Research Organization, The Volcani Center, Rishon LeZion, 7528809, Israel

## Abstract

*Varroa destructor* infestation of *Apis mellifera* colonies carries and/or promotes replication of honey bee viruses like the Deformed wing virus, the Varroa destructor virus-1, the Acute bee paralysis virus, the Israeli acute bee paralysis virus and the Kashmir bee virus that have been well described and characterized; but viruses exclusively associated with *Varroa* were not found. To look for viruses that may associate with- or infect *V. destructor* we performed deep sequencing (RNA-seq) of RNA extracted from honey bees and mites in *Varroa*-infested untreated colonies. Comparative bioinformatic analysis of the two separate contig-assemblies generated from the sequences’ reads annotated using Blastx enabled identification of new viruses unique to *Varroa* and absent in *A. mellifera*: an Iflavirus and a virus with homology to Ixodes scapularis associated virus 2, that we named Varroa destructor virus 2 (VDV-2) and 3(VDV-3), respectively. We validated these findings sequencing the mite- and honey bee-viromes and in separate mites and honey bees randomly sampled. The complete genomes of VDV-2 and VDV-3 bear 9576 nucleotides and 4202 nucleotides, respectively. Phylogenetic analysis of VDV-3 suggests that it belongs to a new group of viruses. Our results open venues for investigating the pathogenicity of these *V. destructor* viruses.

The ectoparasite *Varroa destructor* spread throughout the world, from the Asian honey bee *Apis ceranae* to the European honey bee *Apis mellifera*, since the middle of the last century^1^. The expansion of *Varroa* to new *A. mellifera* colonies broadened its interaction with honey bee viruses^2^ since the mite is able to vector Acute bee paralysis virus (ABPV)^3^, Deformed wing virus (DWV)^4^,^5^, Israeli acute bee paralysis virus (IAPV)^6^, Kashmir Bee Virus (KBV)^7^ and Sacbrood Virus (SBV)^8^. Moreover, *Varroa* infestation deeply changed the nature of DWV infections from common mild asymptomatic infections to symptomatic lethal infections by promoting the selection of virulent DWV strains^9^,^10^,^11^,^12^ and boosting a worldwide viral epidemic^13^. Agricultural production heavily relies on *A. mellifera*-mediated pollination^14^, estimated in 2007 to add globally around $40 billion to crop value annually^15^. Because *Varroa* infestations and virus infections (mainly DWV) became major drivers of collapse of honey bee colonies^1^,^5^,^16^,^17^,^18^,^19^ they therefore threaten food security.

It is known very little about viruses infectious to *V. destructor*. So far the honey bee viruses vectored by *V. destructor* seem not to cause pathogenic symptoms to the mite, including a virus discovered in the mite, the Varroa destructor virus-1 (VDV-1) that is highly homologous to DWV^20^. Also, fragments of a baculovirus were identified by surveying the genome of *Varroa*^21^. Another study identified a Tymoviridae-like virus in Varroa transcriptomes^22^.

To investigate if there are viruses that may specifically associate\infect *V. destructor* we performed deep sequencing (RNA-seq) and comparative bioinformatics analysis of RNA extracted from honey bees in *Varroa*-infested untreated colonies and from *V. destructor*.

Our data revealed novel viruses present in *V. destructor* and absent in *A. mellifera*. The discovery these new viruses paves the way for studying their pathogenicity to *Varroa* and exploring their suitability as means to control this pest.

## Results

### Metagenomic analysis of *A. mellifera* and *V. destructor*

To investigate the viral population (load) in *A. mellifera* infested with *V. destructor* we extracted RNA from honey bees and *Varroa* pooled from four colonies that were maintained without treatment against this mite (see Methods). High throughput sequencing of the libraries prepared from these RNAs resulted in 30,500,539 paired-end reads of 100 bp for the *V. destructor* library and 31,221,496 paired-end reads of 100 bp for the *A. mellifera* library. After removal of adaptor sequences and low quality reads we generated two separate assemblies, the *V. destructor* assembly that had 37,704 contigs with N50 of 824 bp and the *A. mellifera* bee assembly that had 17,274 contigs with N50 of 416 bp (see Methods). The contigs were annotated against the nr-database at NCBI^23^ using Blastx. This analysis identified contigs homologous to most common honey bee viruses like ABPV, Black Queen cell virus (BQCV), DWV, IAPV, Lake Sinai virus1 (LSV1), SBV and VDV-1; some similar to Slow bee paralysis virus (SBPV), Varroa destructor Macula-like virus (VdMLV); and some homologous to bird-, insect- and plant-viruses (Tables 1 and 2, and Supplementary Tables S6 and S7).

**Figure 1:**
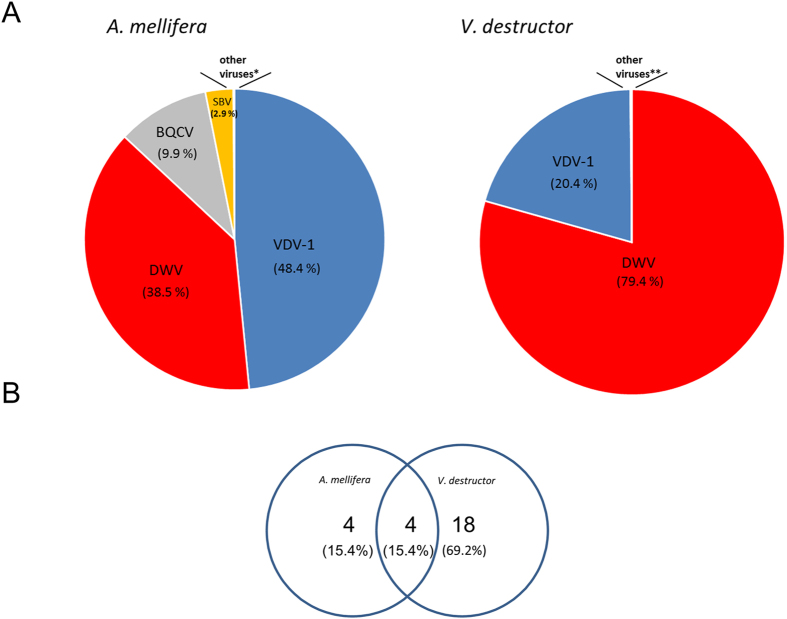
(**A**) Contigs of viruses in *A. mellifera* and *Varroa destructor* in *Varroa*-infested untreated colonies. (**B**) Viruses common and uncommon to *A. mellifera* and *Varroa destructor* (overlapping and not overlapping circles, respectively). Numbers of viruses indicated in the circles, parenthesis % of viruses from total number of viruses found. *and **other viruses specified in Tables 1 and 2.

Mapping of the libraries’ reads to the viral contigs in *A. mellifera* and *V. destructor* resulted in 86.9% and 99.8% of the viral reads respectively that corresponded to DWV plus VDV-1 viruses (Fig. 1A). When we used a cutoff value of 0.0001% of the reads that mapped to the viral contigs we found four viruses that were common to *A. mellifera* and *V. destructor*, and 18 viruses that were unique to *Varroa* (Fig. 1B).

From the contigs found in the *V. destructor* library we further investigated two sets of large contigs with highly significant e values that suggested the presence of two viruses unique to *Varroa*:

1. Contigs that displayed high homology (46% with an e value of <1e^−85^) to the polyprotein of the Iflavirus Brevicoryne brassicae picorna-like virus^24^ (Table 2 and Supplementary Table S7) and,
2. A contig of 4169 nucleotides virus with high similarity (49% and e value of <1e^−50^) to *Ixodes scapularis* virus type 2 (ISAV-2)^25^ (Table 2 and Supplementary Table S7).

Firstly, to identify the Iflavirus from above we designed various sets of primers based on overlapping contigs and amplified the sequences from cDNAs prepared from the original RNA material that was used for generation of the NGS libraries (see Methods and Supplementary Table S1). Classic sequencing of the recovered fragments and further analysis enabled us to elucidate the sequence of a large continuous open reading frame (see Methods and Supplementary Fig. S2A). Furthermore, we isolated viruses from pools of *Varroa* mites. RNA extracted from the viral pellets served to perform virome analysis (for details see Methods) through NGS and bioinformatics as before. This analysis yielded a large contig of 5632 nucleotides that enabled identification of the 5′ end of the viral genome (mapping of the transcriptome and virome reads to the VDV-2 contigs is described in Methods, NGS libraries). The 3′ end of the viral genome was determined by 3′ RACE (see Methods). Thus, we obtained the complete genome of this virus of 9576 nucleotides that we named Varroa destructor virus -2 (VDV-2) (accession number KX578271 and Supplementary Table S4).

**Figure 2:**
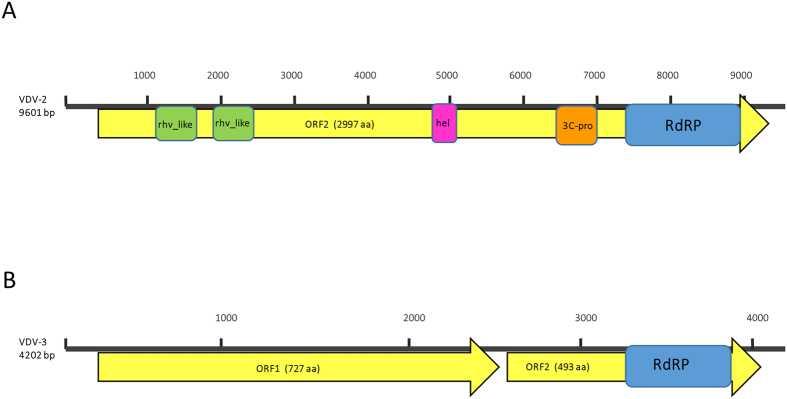
Genomic organization of VDV2 and VDV-3 (**A and B**, respectively). Nucleotide lengths are indicated on the left. Yellow arrows: predicted open reading frames (ORF) with their amino acid length. Colored boxes: identified protein motifs.

**Figure 3:**
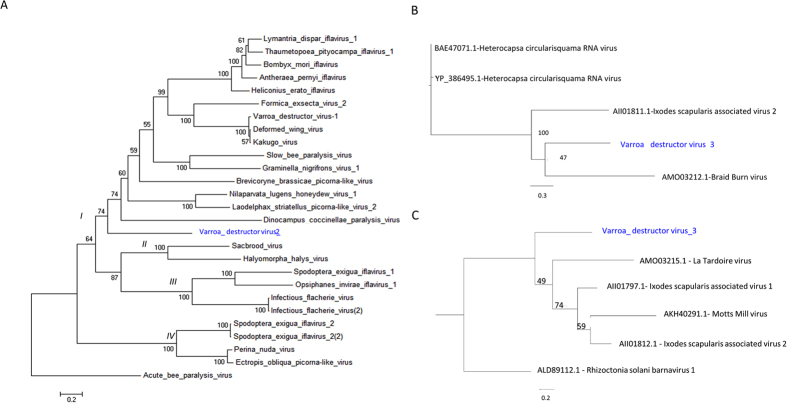
Maximum-likelihood phylogenetic tree based on. (**A**) The complete ORF polyprotein of VDV-2 and Iflaviruses. External reference ABPV. Accession numbers provided in Supplementary Table S3. Background colours indicate the various groups. (**B and C**) ORF 1 and ORF 2 of VDV-3, respectively.

VDV-2 codes for a predicted large open reading frame (ORF) of 2997 amino acids and Blastx analysis revealed that it bears rhino virus-like (rhv), helicase, 3C protease and RNA-dependent RNA polymerase motifs characteristic of Iflaviruses (Fig. 2A). Phylogenetic analysis of the complete genome’s deduced polyprotein amino acid sequence shows the genetic relationship between VDV-2 and other Iflaviruses (Fig. 3A). The phylogenetic tree exhibits four major groups: (branch *I*), the group of Deformed wing virus + Varroa destructor-1 (DWV + VDV-1), branch *II* the Sacbrood virus group (SBV), branch *III* the Infectious flacherie virus group (IFV), and branch *IV* that bears Ecrotopis obliqua virus (EoV) and Perina nuda virus (PnV) as well as Spodoptera exigua iflavirus 2 (SeIV2). Our new discovered Varroa destructor virus-2 (VDV-2) is located in branch *I* which indicates that it is genetically closer to the DWV + VDV-1 group and more distant from branch *IV* of the tree that contains EoV + PnV and SeIV2 (Fig. 3A, *ibid*).

Secondly, the *Ixodes scapularis*-like genomic sequence was recovered using 5′ and 3′ terminal primers designed from the respective contig (see Methods and Supplementary Table S2) that were amplified and sequenced. Further analysis from sequences derived from the *Varroa* virome (described above and in Methods) extended the genomic sequence to 4202 nucleotides corresponding to a novel virus that we named VDV-3 (accession number KX578272 and Supplementary Table S5; mapping of the transcriptome and virome reads to the VDV-3 contigs is described in Methods, NGS libraries). The genome of VDV-3 revealed the presence of two putative major ORFs 1 and 2 of 727 and 493 amino acids, respectively (Fig. 2B). Conserved motif analysis showed that ORF 2 of VDV-3 bore ATPase and canonical RNA-dependent RNA polymerase motifs (Fig. 2B). 3′ RACE analysis indicated that were not polyadenylated residues at the 3′ end of VDV-3. Interestingly, the 3′ terminal 56 nt sequences of VDV-3 (positions 4146–4202) were identical to a portion of the *Bacillus thuringiensis chiA* gene for exochitinase, strains SBS-BT6 and BT3, as well as highly similar to the16S rRNA gene from several Gram positive bacteria (accession numbers HG792452.1, HG792451.1 and AM940962).

Phylogenetic analysis using ORF1 and ORF2 data strongly indicated that this virus may form a new group of positive-sense single-stranded RNA viruses closely related to positive-sense single-stranded RNA unclassified viruses, such as *Ixodes scapularis* associated viruses 1 and 2 and *Drosophila* viruses (La Tardoire virus, Braid Burn virus and Motts Mill virus^26^), and that it may be a far relative of Dinornaviruses (Heterocapsa circularisquama RNA virus) or Barnaviruses as well (Fig. 3B and C).

### Validation of the viruses

To validate our findings we screened individual- and pools of-mites from various colonies for the presence of VDV-2 and VDV-3 using RT-PCR with primers specific for each virus (see Methods).

**Figure 4:**
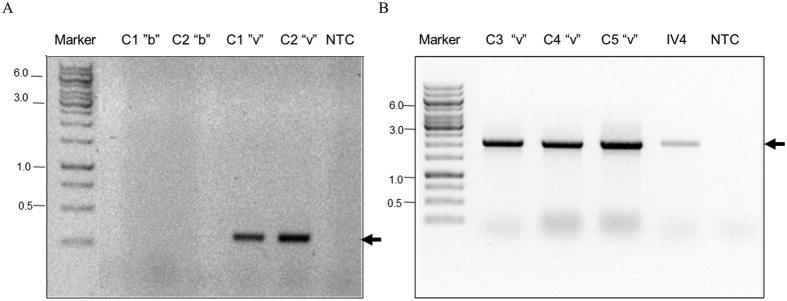
VDV-2 is present only in *V. destructor* and not in *A. mellifera*. (**A**) Detection of VDV-2 by RT-PCR in bees (b) and mites (v) from colonies 1 and 2 (C1 and C2, respectively). (**B**) Detection of the virus in *Varroa* from various colonies (C3 to C5 “v”). IV4, library from original RNA used for RNA-seq. VDV-2 specific primers in A were 5544F and 5879R, and in B, 3777F and 5879R (see Supplementary Table S1). Arrows VDV-2. Marker GeneRuler 1 kb DNA Ladder (Thermo Scientific Inc.).

VDV-2 was found in mites from colonies infested with *Varroa* but not in *A. melifera* bees from the same colonies (Fig. 4A and B, lanes C1 to C5 “v”, and Fig. 4A, lanes C1 and C2 “b”, respectively). Similarly, it was present in the *Varroa* virome but not in the honey bee virome, as well as in a pool of randomly collected mites (Supplementary Figs S1 and S2).

**Figure 5:**
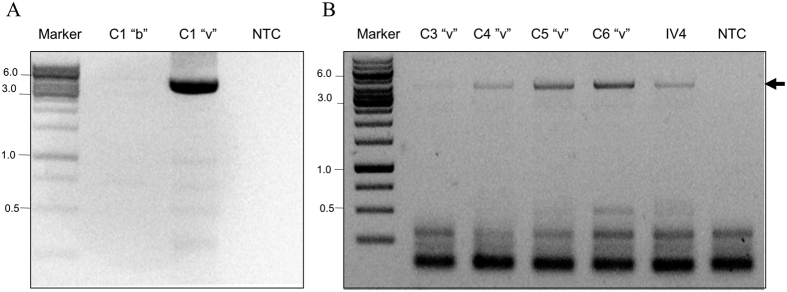
VDV-3 is present only in *V. destructor* and not in *A. mellifera*. (**A**) Detection of VDV-3 by RT-PCR in bees (b) and mites (v) from colony 1 (C1 “b” and C1”v”, respectively). (**B**) Detection of the virus in *Varroa* from various colonies (C3 to C6 “v”). IV4, library from original RNA used for RNA-seq. Arrows VDV-3 amplified with primers 94F and 4145R (see Supplementary Table S2). Marker: GeneRuler 1 kb DNA Ladder (Thermo Scientific Inc.).

VDV-3 was detected only in *Varroa* mites and not in *A. mellifera* (Fig. 5A and B, lanes C1 “v”, and C3-C6 “v”, respectively; and Fig. 5A, lane C1 “b”). In addition, it was present in the *Varroa* virome but not in the honey bee virome (Supplementary Figs S3 and S4).

Interestingly, VDV-2 was not found in all the mites analyzed (Fig. S2A and B, lanes 7, 15 and 16 “v”, respectively). The same was valid for VDV-3 (Fig. S4A and B, lanes 1, 9–11 and 13–16 “v”, respectively).

## Discussion and Conclusions

Most of the studies performed focused on viruses transmitted by *Varroa destructor* to *Apis mellifera* and very little is known about viruses of this mite. A genomic survey of the *V. destructor* genome identified fragments of a novel virus related to the Baculoviridae but there were no reports about complete identification of the virus^21^. A Bee Macula-like virus (BeeMLV) with similarity to viruses of the Tymoviridae family, with a poly-adenylated ss RNA genome of about 6500 nucleotides, was detected in *A. mellifera* and *V. destructor* and another Tymoviridae-like virus named Varroa Tymo-like virus (VTLV) was found in *Varroa* transcriptomes^22^. BeeMLV and VTLV shared about 50% amino acid identity for the polyprotein and capsid proteins. Interestingly, BeeMLV showed high prevalence in *V. destructor* and the analysis indicated that while honey bees were hosts the mites were most likely vectors^22^.

Our metagenomic analysis of the *V. destructor* transcriptome that concentrated on viruses associated with the mite and compared them to viruses associated with honey bees from the same colonies enabled the discovery of viruses exclusive to *Varroa* and their characterization. Thus we found, the Iflavirus VDV-2, and VDV-3 a virus with some similarity to the Ixodes scapularis associated viruses 1 and 2^25^. Moreover, we validated the presence of these viruses in *Varroa* mites from randomly tested colonies as well as in the mite virome and confirmed their absence in *A. mellifera* samples and virome.

Recently a classification for Iflaviruses was proposed dividing the tree in four types^27^. Type I including the DWV, VDV-1, Brevicoryne brassicae virus (BrBV) and Lymantria dispar iflavirus (LdIV)-group of viruses, Type II including SBV, type III including Infectious flacherie virus (IFV) and type IV including Spodoptera exigua iflavirus (SeIV) and Perina nuda picorna-like virus (PrPV)^28^. Our phylogenetic analysis attributes branches *I II, III* and *IV* to Types I, II, II and IV respectively, in accordance with the above published division and suggests that VDV-2 belongs to Type I Iflaviruses.

A recent work identified two viruses, Ixodes scapularis associated viruses 1 and 2, in the tick *Ixodes scapularis*, with partialy genomic sequences of 2.8 kb and 2.3 kb, respectively that bore RdRP motifs^25^. We discovered VDV-3 with 49% similarity by Blastx to ISAV-2 and identified the viral genome of 4202 nucleotides bearing two putative ORFs, ORF1 and ORF2. ORF2 bears ATPase and RdRP motifs. The genome of VDV-3 is not polyadenylated. The 3′ terminal 56 nt sequences of VDV-3 were identical to a portion of the exochitinase *chiA* gene of *Bacillus thuringiensis* and the 16S rRNA gene from several Gram positive bacteria. Further studies focused in the replication of the virus will be needed to elucidate the role of these sequences in the VDV-3 genome.

The finding that both viruses VDV-2 and VDV-3 were exclusively associated with *Varroa* suggests that their localization and or replication occurred in tissues of the mite that were not associated with sucking hemolymph of the honey bee in contrast with viruses vectored by *Varroa* to *A. mellifera*. Not all the mites tested were positive for VDV-2 and VDV-3, and some mites bore one virus but not the other suggesting that the rate of infection (or prevalence) was different among hives and even between mites in the same hive. In addition, it is possible that the mite bears the viruses and they were not replicating in the mite or they replicated well in some mite but less good in others. More experimentation, including specific detection of their negative-sense RNA strands and quantification is needed to proof these hypotheses.

Our results provide new tools and open new ways for investigating viruses of *Varroa destructor*, their pathogenicity to the various developmental stages of the mite as well as their distribution in mites that spread to honey bee colonies worldwide. This could pave the way for their future utilization in mite control since *A. mellifera* seems not to be carrying the virus/es.

Moreover, our findings contribute as well to develop further tools to identify viruses of ticks that are vectors of a wide range of human and animal diseases^25^.

## Methods

Honey bee *A. mellifera* colonies (*A. mellifera liguistica*) used in the study were transferred from the experimental apiary of the Agricultural Research Organization located in Zrifin to the Institute of Plant Protection at the Volcani Center, Israel. The colonies were maintained without treatment against *Varroa* for a period of 30 months before sampling but they received seasonal sugar feeding and Fumagilin treatment against *Nosema*.

Transcriptome- Alive *Varroa* mites and honey bees were collected from February to May 2013, from a retractable tray under a screen net at the bottom of the hive. A pool of 117 mites, and 5 worker bees per colony were collected from colonies 3, 4, 8; and 193 mites and 15 worker bees were collected from colony 23.

Virome: Alive *Varroa* mites and honey bees were collected from October 15 to February 2016. 32 bees in total from hives 1, 3, 5 and 23 (4 bees per hive), 14 (6 bees), 81 (9 bees) and 401 (1 bee) and 606 mites from the same hives (85 of them from emerging bees and the rest from free falling mites as described above).

Validation- worker bees and mites were randomly collected from four hives 7, 6, 10 and 401 from June to July 2016.

The honey bees and mites were frozen immediately at −80 °C until RNA extraction.

### RNA extraction

RNA was extracted from the samples described above from four colonies and subsequently pooled (30 bees and 310 mites for the *A. mellifera* and the *V. destructor* cDNA libraries, respectively) using TRI reagent^®^ (Sigma-Aldrich) according to the manufacturer’s instructions in a Geno/grinder homogenizer (Metuchen, NJ, USA). The extracted RNA was further purified by precipitation with 2.5 M Lithium Chloride. The samples were washed in 75% ethanol, dissolved in RNase-free sterile water and stored at −80 °C until they were used. The quality and quantity of the extracted RNA was evaluated using an Agilent Bioanalyzer (Agilent Technologies) using a RNA 6000 Nano LabChip kit. RNA with values of RIN (RNA Integrity Number) bigger than 8 was used for the preparation of the libraries.

### Virome preparations

32 honey bees and 600 *Varroa* mites from 7 honey bee colonies were ground separately to homogeneity in sodium phosphate (0.01 M, pH 7 containing 0.02% Sodium diethyldithiocarbamate). The tissue debris of each sample was clarified by 10 min centrifugation at 800 g at 4 °C in Hettich Universal 32R centrifuge (Hettich Lab Technology, Germany), followed by a second 15 min centrifugation at 14,600 g at 4 °C using a SelectSpin 17R microcentrifuge (Select BioProducts, New Jersey). The supernatants were overlaid to a solution of 30% sucrose in phosphate-buffered saline pH 7.5 (PBS) and subjected to 4 hours ultracentrifugation at 100000 g at 4 °C in a Sorvall Discovery 90SE ultracentrifuge using a TY35 rotor (Hitachi). The viral pellet was re-suspended in PBS and utilized for RNA extraction as described above.

### Library construction and NGS

Libraries construction from the above RNA pools and NGS (RNAseq) were performed at the Technion Genome Center (Technion, Israel Institute of Technology, Haifa) in the HiSEq 2000 platform (Illumina). Paired-end sequencing (100 bp) resulted in 30,500,539 paired-end reads for *Varroa destructor* library and 31,221,496 paired-end for the *Apis mellifera* library. In addition two libraries of 125 bp paired-end reads of virome from *V. destructor* and *A. mellifera* were sequenced at the The Nancy & Stephen Grand Israel National Center for Personalized Medicine in the Weizmann institute of Science with Hiseq 2500, resulting in 80,000,000 paired-end reads and 71,473,693 paired-end reads for *Varroa* and honey bee libraries, respectively. The transcriptome and virome files of *V. destructor and A. mellifera* were uploaded to SRA database under accession numbers PRJNA329427 and PRJNA329428, respectively. The sequences were cleaned from remains of adaptor sequences and low quality reads using Trimomatic software^29^ and assembled *de novo* using Trinity^30^. The assembled contigs were subsequently translated and aligned to the GenBank nr database (all nr database without filtering) by Blastx^23^ (with a cut-off value of <1e^−5^).

Two sets of contigs, one with homology to Iflaviruses and the other homologous to Ixodes scapularis associated virus-2 were further investigated (later designed VDV-2 and VDV-3). 3590 reads of the *Varroa* transcriptome were mapped to VDV-2 out of 30500539 that make 0.011% of the reads and 738 reads were mapped to VDV-3 virus out of 30500539, which makes 0.0024% of the reads (displayed in Supplementary Figs S5A and S6A). NCBI CDD (conserved domains) program was used to detect domains found on the ORF^31^.

By using 5′ and 3′-terminal primers we were able to recover two large fragments of the Iflavirus-like virus genome (see Supplementary Table 1).

Virome reads were assembled using Trinity as described above. From the *V. destructor* virome 65,734 contigs were generated with N50 of 549 bp, we used Blastx against the NCBI non-redundant database to annotate the contigs. From the virome of *A. mellifera* 11,997 contigs were generated with N50 of 428 bp In the virome 2329 reads (0.0029%) mapped to VDV-2 while 8264 reads (0.01%) mapped to VDV-3 out of 80000000 reads in total. Mapping of the reads to VDV-2 and VDV-3 is displayed in Supplementary Figs S5B and S6D; visualization of the read maps was performed using Integrative Genomics Viewer, IGV^32^.

### RT-PCR

cDNA was prepared using Maxima-Reverse transcriptase (Fermentas-Thermo Fisher Scientific, Burlington, Canada) using oligo-dT and random primers according to the manufacturer’s instructions. For screening purposes 100 ng template RNA were used. A second re-screening with 2000 ng of the same RNA was performed for honey bee samples. RT-conditions: incubation of RNA and primers at 65 °C 5 min, following by addition of buffer containing 50 mM Tris-HCl (pH 8.3), 75 mM KCl, 2 mM MgCl_2_, 5 mM DTT, 4 units of RNase inhibitor Ribolock^®^ (Thermo Scientific™), the RT enzyme (200 units) in a 25 μl volume, and further incubation at 55 °C for 30 min. The reaction was terminated by heating at 85 °C for 5 min.

PCR amplifications were performed using LongAmp Taq Polymerase (New England Biolabs) and specific primers (see Supplementary Tables S1 and S2) in a BioER GenePro TC-E-96G apparatus (Hangzhou bori Technology Co., Ltd, P.R. China). For long templates amplification 1–2 μl cDNA were used with 2.5 units of Taq DNA polymerase with appropriate buffer, 1 mM dNTP, 2 mM MgCl_2_ and 0.2 μM of each forward and reverse primer in a final reaction volume of 10 μl. The protocol used was 94 °C for 2 min, then 38 cycles of 94 °C for 20 sec., 57 °C for 40 sec., 65 °C 4.5 min. and a final extension step of 65 °C for 10 min. PCR-validations were performed with GoTaq^®^ (Promega Corporation, USA) using 1–2 μl DNA template and 0.2 μM of each forward and reverse primer in a 15 μl reaction with the following conditions: 95  °C for 4 min., 32 cycles at 94 °C for 30 sec, then 58 °C (for VDV-2) or 55 °C (for VDV-3) for 30 sec., 72 °C for 2.5 min. (for VDV-2) or 2 min. (for VDV-3) and a final extension step of 72 °C for 10 min.

Amplification and validation primers are provided in Supplementary Tables S1 and S2 and in the corresponding legends of the figures. All the PCR reactions included non-template controls (NTC). PCR products were evaluated by conventional agarose electrophoresis.

### Sequencing and 3′RACE

Conventional sequencing from PCR amplicons or cloned DNA fragments in the vector pTZ57R/T (Fermentas-Thermo Fisher Scientific, Burlington, Canada) was performed at the Biological Services Unit of the Weizmann Institute of Science, Israel. 3′ RACE was performed using the SMARTer RACE 5′/3′ Kit (Takara) according to the instructions of the manufacturer.

In brief, cDNA was prepared with the SMARTer RACE 5′/3′ Kit using 1 μg of RNA from the viral fraction in a solution with the kit components 3′-cds primer A, first strand buffer, with 2 mM dNTPs, 10 mM DTT, RNase inhibitor 0.5 μl and SmartScribe RT at 42 °C for 90 min., followed by 70 °C at 10 min. The cDNA obtained was diluted with 10 μl of Tricine-EDTA buffer (supplied by Takara) and 2.5 μl of it were used in a solution of 3′-RACE Seq. buffer with SeqAmp DNA Polymerase provided in the kit and the RACE-specific virus primer (information provided in Supplementary Tables S1 and S2 for VDV-2 and VDV-3 RACE primers). PCR amplification was performed at 94 °C for 30 sec. then 72 °C for 3 min (5 cycles), followed by 94 °C for 30 sec., 70 °C for 30 sec. and 72 °C for 3 min. (5 cycles) and finally 94 °C for 30 sec., 68 °C for 30 sec. and 72 °C for 3 min. (25 cycles).

Amplicon and Sequencing primers are shown in Supplementary Tables S1 and S2. The complete sequences of VDV-2 and VDV-3 are provided in Supplementary Tables S4 and S5, respectively.

### Molecular Phylogenetic analysis

The complete predicted ORF of VDV-2 and ORF 2 of VDV-3 were used for the analysis. The former was compared against the complete ORFs of the Iflaviruses (Supplementary Table S3).

The VDV-3 ORF 2 was compared against Ixodes scapularis associated viruses, *Drosophila* viruses, Dinornaviruses (Heterocapsa circularisquama RNA virus) or Barnaviruses (virus names and accession numbers are provided in Fig. 3B and C). The alignment was performed with Muscle alignment software^33^.

The evolutionary history was inferred by using the Maximum Likelihood method based on the JTT matrix-based model^34^. The bootstrap consensus tree inferred from 1000 replicates^35^ is taken to represent the evolutionary history of the taxa analyzed^35^. Branches corresponding to partitions reproduced in less than 50% bootstrap replicates are collapsed. The percentage of replicate trees in which the associated taxa clustered together in the bootstrap test (1000 replicates) are shown next to the branches^35^. Initial tree for the heuristic search was obtained automatically by applying Neighbor-Join and BioNJ algorithms to a matrix of pairwise distances estimated using a JTT model, and then selecting the topology with superior log likelihood value. Evolutionary analyses were conducted in MEGA6^36^.

## Additional Information

**How to cite this article**: Levin, S. *et al*. Two novel viruses associated with the *Apis mellifera* pathogenic mite *Varroa destructor. Sci. Rep.* **6**, 37710; doi: 10.1038/srep37710 (2016).

**Publisher's note:** Springer Nature remains neutral with regard to jurisdictional claims in published maps and institutional affiliations.

## Supplementary Material

Supplementary Information

## Figures and Tables

**Table 1:** Percentage of total viral reads mapping to viral contigs of the *Apis mellifera* library, cutoff at 0.0001%.

Virus	Reads (%)
Varroa destructor virus-1	48.4452
Deformed wing virus	38.4967
Black queen cell virus	9.9200
Sacbrood virus	2.9849
Grapevine Bulgarian latent like	0.0193
Lake Sinai virus 1	0.0035
Tobamovirus	0.0032
Farmington virus*	0.0001

^*^A new virus of the length 14,606 bp was discovered in the *A. mellifera* transcriptome.

The virus belongs to the genus of unclassified Rhabdoviridae with protein similarities to Farmington virus, Drosophila sturtevani rhabdovirus 1, Wuhan House Fly Virus 2 and Drosophila subobscura rhabdovirus (see also Supplementary Table S6).

**Table 2:** Percentage of total viral reads mapping to viral contigs of the *Varroa destructor* library, cutoff at 0.0001%.

Virus	Reads (%)
Deformed wing virus	79.371
Varroa destructor virus-1	20.457
Brevicoryne brassicae picorna-like virus	0.0711
Bat feces associated picorna-like virus	0.0291
Acute bee paralysis virus	0.0162
Halyomorpha halys virus	0.0138
Sacbrood virus	0.0086
Black queen cell virus	0.0073
Varroa destructor Macula-like virus	0.0063
Dragonfly Cyclovirus	0.0054
Slow bee paralysis virus	0.0031
Ixodes scapularis associated virus 2	0.0028
Formica exsecta virus	0.0020
Spodoptera exigua iflavirus 1	0.0010
Heliconius erato iflavirus	0.0010
Aransas Bay virus	0.0009
Jos virus	0.0008
Israeli acute paralysis virus	0.0008
Epiphyas postvittana nucleopolyhedrovirus	0.0005
Dhori virus	0.0004
Antheraea pernyi iflavirus	0.0003
Anguilla anguilla circovirus	0.0001
