# Supplementary material for: Two novel viruses associated with the Apis mellifera pathogenic mite Varroa destructor
Source: Sci Rep. 2016 Nov 24;6:37710. doi: 10.1038/srep37710 (PMC5121581; doi:10.1038/srep37710)
Supplement: Supplementary Information [file srep37710-s1.pdf]

Supplementary information

Two novel viruses associated with the *Apis mellifera* pathogen mite *Varroa destructor*

Sofia Levin[1], Noa Sela[2] and Nor Chejanovsky[1]

[1]Entomology Department and [2]Department of Plant Pathology and Weed Research, Institute of Plant Protection, Agricultural Research Organization, Israel

Table S1- Primers used for amplification, sequencing and 3'RACE of VDV-2 from the *Varroa destructor* library

Primer name	Sequence	Application
1605F	TTTGCTCACTTTAACAATCACAAGT	amplicon a- 4274bp
5879R	TTCCTCTCCAGCTTTAGGTGCT	amplicon-a- 4274bp
5822F	AGAAATTTAGGGACCTCTCCGA	amplicon d- 3218bp
9040R	TGCGTCCTAAGCCAATTACG	amplicon d- 3218bp
8775F	<u>GATTACGCCAAGCTT</u>CGAATCGCGTGGA TGGCCATTAT	3`prime RACE
2237F	TGGGTATGACCGTGCTAGTG	sequencing
2926F	GTGGGTGGCGGTATAATGGTA	sequencing
3777F	GCAACATCCCGTAACAATGG	sequencing
5544F	GGAATTGCGTCGAATTGTCAC	sequencing
5879R	TTCCTCTCCAGCTTTAGGTGCT	sequencing
6555F	TGTGGCGTTTACTTGGGTGA	sequencing
4416R	CAAGCACACTATCAACCACCG	sequencing
5213R	CGGAGGAGCTACATGCATACC	sequencing
8385R	TGGTGTATTGTCGCTGCTGA	sequencing

Primers sequences presented from 5' to 3'. The numbers correspond to the nucleotide number in the viral genome. F, forward; R, reverse. Two pairs of primers were used to amplify two large PCR fragments from the *V. destructor* library resulting in overlapping amplicons a and d. Underlined sequences added for 3' RACE (according to manufacturer Instructions). 5`prime is based on a contig of 3879 nucleotide length obtained from the *Varroa destructor* virome analysis the contig's sequence 3`end overlaps the 5`prime end of amplicon a.

Table S2- Primers used for amplification and sequencing of VDV-3 from the *Varroa destructor* library

Primer name	Sequence	Application
94F	ATAGGAATGGCGGTCGTAGC	amplicon
4145R	CACGACGGACATACCACTTG	amplicon
727F	ACAGCCGCTCGTATCAAGAC	sequencing
1602F	GCCTTGGTCGAAGACTTGAA	sequencing
3369R	TTAGCCAACCAACGAAAACC	sequencing
4091F	GATTACGCCAAGCTTCCTGGGAGTGGAACCTTGCCACTAGG	3`prime RACE

Primers sequences presented from 5' to 3'. The numbers correspond to the nucleotide number in the viral genome. F, forward; R, reverse. Underlined sequences added for 3' RACE (according to manufacturer Instructions).

Table S3- Accession numbers of viral sequences used for Iflaviruses' phylogenetic analysis

Iflavirus	Accession number
Acute bee paralysis virus	NP_066241.1
Antheraea pernyi iflavirus	AHI87751.1
Bombyx mori iflavirus	BAS18834.1
Brevicoryne brassicae picorna-like virus	ABP57198.1
Deformed wing virus	AMK01489.1
Dinocampus coccinellae paralysis virus	AIM39350.1
Ectropis obliqua picorna-like virus	AAQ64627.1
Formica exsecta virus 2	AHB62422.1
Graminella nigrifrons virus 1	AJT58559.1
Halyomorpha halys virus	AGY34702.1
Heliconius erato iflavirus	AHW98099.1
Infectious flacherie virus	ADP24157.1
Infectious flacherie virus	BAA25371.1
Kakugo virus	BAD06930.1
Laodelphax striatellus picorna-like virus 2	AIX94679.1
Lymantria dispar iflavirus 1	AIF75200.1
Nilaparvata lugens honeydew virus 1	BAN19725.1
Opsiphanes invirae iflavirus 1	AKN81079.1
Perina nuda virus polyprotein	AAL06289.1
Sacbrood virus	AND80528.1
Slow bee paralysis virus	ADI46683.1
Spodoptera exigua iflavirus 1	AET36829.1
Spodoptera exigua iflavirus 2	AFQ98017.1

Spodoptera exigua iflavirus 2	AHX00961.1
Thaumetopoea pityocampa iflavirus 1	AJC98140.1
Varroa destructor virus-1	AGO86045.1

Table S4- Complete VDV-2 sequence- 9576 nucleotides

AGCGGCTACAAAGTAGACCGCTCCGTCCAGTTTTATACGAAGCCGAGGCCCGGCAC
AACTGCGAAGGGGAGGTCCCCCATATTGCGAAGTGGTAGCCCCACAAATTTTAATA
GTAATTTGTTTAAATATATTAACTTTAAGTAAGGATTTAATTTTAATATTCAAATAG
ATATACCCTTTAGGCCTTTTGGCTGAAGGGTTACAAAGGCCAAGTGCCTTAAGGTGA
GCCCATAGGGCAGTTAAATTTTATTTTATTTGCTATATACACATATTTTAGTTTTGAT
AGTCCCTTAAGGACTATTTAGATAGATTATATATTATATATAAATTAGTTAGTATTTT
GTTACATATTATATATTTATATTTTATATTATATATATATTAAAATATTAATTATGAT
TATTTATAGTAAATTACGTAGTGCCCCGGACACATGGCAAAAAGTCCGGGGTTTTAA
TCCCCGTAGGCAGTGGGAAAACTGCCGACAGAATAGATTGGACCGCATAGCGGCGG
TTCAGCTTAACGTAACTGCAGTAGACCGGCAGTTACGAAAATTGGAGGGCATTTCA
AATGCCCACCTGATTTCAGCCCAAAAGGGCAATTCCCGTGCTAGGGAGGAAAGGGC
AAAAGCCCTTAAACAAATAGATGTTCTCAGGGAGGACAAGGTGCGCCATCAGGCCA
CCATTACAGAACTCTCGAAAGAGACTCGTCGAGAGTTCAAAATGCGCAAGATTTAT
ATGCATCGCTTGCGCGAGGAGCGCCGTGAGGTGAAGGCGCGAAAGGTTGCTAAGAG
CAACCAATTTGCGGTCCTTAAAGAAGAAAAGGACCCTTATTCATCCAGGTGGGTTA
CACCTGATGAATATGTCGCTGAGCAGCGACTTTTTAAGCGCATTGAAAGATCTAATG
CGCTTTATTTTAAGCGCAAAGCGCTTAAGGAGGAGGCCAAACAACAGCGCCTCGAG
AGGCTCCTCAATGAGGAACCTCAATATCATCCACAGGTGAAACCTGTGGATCGCTCT
GGCAGGCTACCGCTGGAGCAAATATTGCTCAACTTAGGTTGGGTTTGGGACAGGAG
GTCCCATATTATCAGCCGTGATTTTCAATTGCGGCCTTATGGAGCCCAGATGGGCTC
AAAGTATCAATGGGCATATTATCAGCCCATATATTATACAGCCTATAACACTAAGGC
TGTTTTTGTTCCCCAGATGGAACAAGTTGAGGTCCCAAAGATATCTGGACCATCAAA
AAAAGTGCGACTTTTTAGATCCAGTCGTGTTACAAATAAGCCCGCTACGATGGTTCC
TTTGTCTCCACAAATGGATAATCAGGGCGCTCACGGTGAACAGCAATCGGAACAGG
TGCAGAGGAATTCCGATACTTTGGTGGTTTCGGCCACCACTTCTTCTGTTGGGCATC
ATCCACCAATATCAGGTTCAACGGAAGATTTTTGTGGTGATATAAGACCAATCACCG
ATGATACGCTCGCTAGTCGAGCTTTTGTATATCGTATGGGAGAATGGACAACATCTC
AACCAACTAACACTTTGTTGTTCCGTAGCAAACTCCCATGTGATGTTATTGTGGGCT
TTAATGATCAACCTAATTTTGCTCACTTTAACAATCACAAGTATTGGCGTGGGGATA
TTGAAGTTGAACTCCATGTCACATCTAATGCCAGGATGATAGGAAGTTTGCAAACGT
CCTGGTATTATGGATTGGAATGGGACGCCCATCGAGCCCTAAAGGATAATGTATAT
ACTAATTCTCAAACAATGCATTGCCTAGTTGATGCGATGAACAGCAATGTAGGTGTC
TTGAGAATACCATTTAGGTGTCCTAAGTCCTTTTTGGACTTGCAGCGTGTACATATA
GGTGGTAATACAACCATACCGGGTTTGTATTTAGGCACCCTTGATGTACGTGTGCTC
AATCAGTTGGCTGTGGCAAATTCTAATACAGCTAACGTGGCTAATTGGACCATGTCC
ATCAAGTTTGTAAATAATAGGTTTATGGGTCCCGTGCATAGGAAATTACTCATACCA
CAAATGATGGGGGCTTGGATGGCTTATCAAATGTTTAAAGAATTACGTGGTGATGCT
AATAGAGACCAGCCTATTAACCCATCCACGCCCTCTCATATTAAACCTGTGCCTACA
GACGATTGGGCCTCAGGTAATGGGCAATCTTCAACAACTTTCCCATTGCGTTTAAAT
CCCAGTGGTCTGCTTGTGTATAATGGGTATGACCGTGCTAGTGATATTGATGAAATG
AAGGTTGATTTTGTAAAGAGTGTATTTGGCCTTCTTCATGTTGCTAAAAGGTCGGCA
CAGGACACTGTACAAACGTGTATATATAGTATGCCTGTTGACCCAATTCTCCCGCCC
GATATGTATTATAATCATGATTATTTAGGTGAGAATGTATATGCCTTACCACCTGTG
GGTGTTTTGGCTAGTATGTTCTCATTATGGAGGGGAGAGTTGGAGGTGCGTTTAGAT
GTTATAGCTGCGACCGATGTACGTGGGCGTTTAATGCTTGCTTATGTGCCTAGTGAG
AATGAGGTTGAGTATGCAGTGGCCATGGGTTCATCAACTGTATATTATGATATAGAT

GAGAATAGGTCTTTTACATGGACCATTCCGTATATGTCGGATCGCCCATGGCGTATG
AGACGATATCAGCCTGATAGTCCCGGGTTCGTAAAAGACCATGGGAAATTTTATAT
ATTTATAACTAATCGGCTTATTATAAATAATACAGTCCCTGATTTTGCTTATATAAAT
ATATATATTAGAGGAGCCCCTAGTTTTGAAGTTGCAGTACCGTGTCAGCCTTTGATT
GGTTTGGCATGGGAACCAAAGATCAGAACTGGTACCCATCAGCTAGAAATTGGATT
TTTGTCTGGTTATTGGCCAGTATACGTTGGTGGGTGGCGGTATAATGGTAGTAGGGT
AGTGATGCGATATGGCGAGACAACCGATCATATTGCACAATTGGACTATGGGGTTA
TGTCTAAATTCCTTACTGCCAAGGTTAAACGAAATGTACCTGGTTATTTTAAGATTA
AAAGTTTAGATCCATCAGTTCAAACACCTGGTACTAATTATGCGGCCTTTAGGGGGT
TACAGGGCGATTATTTGGCTAAGGGCTTGTTCCCTGTAATAACAAAATCAGGGTATT
GTTACACTATACCTGTGGTCACCGATTCTGATGCGGTTAATTATTACCGTCATCTGA
AGGAAACCCTCTGGGATTTTCCACCCCAGCAGGTACAATATGTTTTTGTTGAAAATT
CAGAATATTGGTGTGCCAAAGGTAATCAAAATTTGGTATTGGAATGGACAGATGTC
GTCTCGCGTGCCGAAATGGATGAGCGTATGAATGTTATAAATGGTGTAAATGAAAA
CTATACAGCTGTTAGGCGCACAGGACATGGTCTTCAATTATTTGGTGAGTCGTTTGG
AGACCTTAAGGATGTTTTAAGGCGGACAACTATGTATATGGCTTGTTCGCAAGATTT
TAGTTCAAGATCAAACCTTGTGGATATTAAATTACCGGTTATTCCGCAAGGTCTTGT
GTTTGATCAAGGGTCTGACGAGAATCCCAATTATTCCTGGAATTGGATTAGGCAGG
GCAATAATCAAATTATTAATAGcGGTTATAGATTCTTTACGGGCaGAATGCATTTTAA
GATTAGGGTGTTTTCAGAAAAAGATAGAGTTGCATTTTTCGCCCAACACCGTCCCCA
TTTAAATTACATAGTaGATAGGCCAACAGCAACATCCCGTAACAATGGTGATAGTTA
TGTTACATTGGAAGAGATGTGGCAACACGGGTATGCCACTATGGTCCAAGATTCGG
CTAATAATAGATTCCTTGAAATTGAGGTACCTTTTTATCAGATGGGCACTTGTGGAA
TAGGTCAAAGGATAGATATGGAGTCTATAGAGTCATATGATCCAGACCTTATCCATT
ATTGTTCATTGGGTGAGTTGGTGCTAGGTTCTTTAGGCGGTAATTCGGCTGCCCCCC
GTACTTATCAAGTATGGAGTCATGTCTCTGATGACTTCAGGCTTTCTACGTTCCAGG
GATTCCCGCAGATGATTTTTTTGTCTTATCAGGATCCTGTTGCTGTCCCCGACGAAAT
TAAATTATTTAAATCCCCAAAAATTTATTATAAAAAAGTAAAGGGGCAGCGTTCTCA
AAAACCCAGTGCGCCTGATTGTGTAGAGTTGAGTGAGTTTAAACCCCAGATGGAGG
TACCAGAAATGTATGTGCCTGTAGTTAGTGACATATTTCATAGTGTTAGAAATGTGT
CCAAAACAAGTGCTACAGTGCGTAATGTTGTGAATAAGATTGATAATATACTTCCA
ACTGGGGAGAGTAGTTTGGCATCGGTGGTTGATAGTGTGCTTGGTAAGTTTAAAGAT
AGTGTTGATTATAATTCTGTACGTATGACATTATCGAATATTATGTCTAATATAGTG
CATTGTCTTATTAACCCGGACCCAAAGACAATCGCTTGGGCCCTTATGTCTATAATA
TATAATATATTCCATATTAAGGGTGTTATTGTTTCTAGGGTTATTGATATTGTGAGGG
GTCTTATTGCTCATCTTGTGAATGTGAGGGAATGTATGAAACCGCAAATGGATGGTG
TGTATGCTGACGTATATGAACCTCTGCAAGGTGCCGATGAGGGATTTGTAAGGAAA
AATATAACAGCACTTTTATCGCTTATTATGGGCGCTGTGTTTACTTATTTAGGGTCTA
GAAAACCTGTTAATTATAATGATTCGTTTGCTTCTAGACTCTATGAGATTTTAATTGG
TTTTAGCAGAAATACAACCCAGCTGGTTAGATTCTTCCAAAACACGATAGAATTATT
TATAGAAATTTTTGACTGGTGTGTAGGAAAAATTTGTCCCAAGTGGGATGCTGTTAG
ATTTTTGGCCTCTAATGATCGTGATCTTTATAAGTGGGCCAAGAGCGTACAATATCT
TACTGACGGGCGTAATCGGAATTTAGTTCTTACAAATCCTAAATTGGTAGCAGAATT
GTATGCTAGTGCGAATGAGGCCTGTTTCATAAGACAGAGGATGTTGGCATTGGATA
AATCTCTTAGTAACGCACAGACCATTACTATAAATAAATTAATTAATGACATCCTTA
CTTTTAGGGATCAGGCGTCAGGTATGCATGTAGCTCCTCCGGTTAAGTTTGAGCCAT
TTGTTTTACAATTAGGTGGTCCTCCTAACATTGGTAAGTCTCATTTGTTGCAGGAGCT
CACTGTTGATTTATTAGAACATGTGGGTTATTCAACATGGGGTGAACCAATTTATAT
TAGGTCTCCTGGTAATGACTATTGGAATGGATTGACCAATCAGCCGATTGTTATTTA
TGATGATTTCCTGGCTGTTACGGCTGATCCAGTTGGGACACAACAAATTTCTGAGTT
GTTCCAACTTAAGACAAGATGTGTCTTCAACCCTCCTATGGCTGCGGTCGAAGATAA
AACTATTAGGTATAATCCAATAGTCGTTGGAATTGCGTCGAATTGTCACTTCTGGAA
TTTGTGTGGTGTTAGTAACCCTGAGGCGGTTTATAGGCGTAGAGACGTATTGATAGA
TGTCGCTCTCAAGGGCTACAAGCAGATTTCAGAGGTGCCTCCGGAGGTGCTAAAGG
AATATGGTCATTTAAAATTTAGGTTTTATTATAATCCTGCTCAGTCTTTAAGCCCGAC
AACAGAATGGCAGGATTATGCTGATTTTAAATTAGAATTGTGTAGAAGGTTTAAAA

ATTATTATGAAAAAGAATCAAAGAAATTTAGGGACCTCTCCGAAAAATTATATAGA
TTAGCACCTAAAGCTGGAGAGGAAGAGTTTAAATTTGCAAATCATGAGGTTGGTGA
GGCATTAGATAAATTCTTTGAGCGTCATAATGTCCTTGCCTCCCATGATGATATAGT
TATTTCTGAGGTTATGAATATGATTAGCGAAAGAGCACGTAATAGGCGGCCTCAGA
TTTATTCTAAAATGGGTGAACTCCTCAAAAAAGGAGAAAATTCTAGAAAAATGACC
GTATCTCAAGCGCTTCTGGAGGCTTATTGTTTAACTAATTCCCCCCCATTAAATCCTG
ATTGTGTCAGAGATAAAATTTACAAAAATTTACGGGATAGATCTCTGATACCCACCA
CTGAAAAGATTAAATTGGCTATGGTTAAGGCTAAATTAGAGGCTCAAATGGAAACA
GAGGAGCCAAGTATGAGTGAGTATATGAGCGTACATGAATACATGTGTCAGGAATG
GTTGATGGCAAGTATGCTTCACTGGGAGAGTGAGAGAAAAGAATGGGTGATTCGTC
TCCATGCAGAATTAGCTGGAGATGATGAACGATTGGAGGCTGTGGCTAAATGGCAG
AAGAAACATAAGATTGTCTATGAAATTGAAAATGCCACCATAAAAATAGACAATAA
TCCATGTGGAAATGGTGTTTGCCCTTGGAGTGAGTCTAATATTAAAGCCAAGTTCCT
TGCTGATTTTATTCAGCTGGGATGTGGCGTTTACTTGGGTGAGTCCAGAGGTGTGGA
CATCATTGAGGCAGGTCTTGTGCCGGAAGACACAGCCGTTGAATGCGTGCCCGCTTT
GGAAGAAATGACTGATGAGACATGGTTGATAAAGGTGCGTAATTTAATATGGAAGG
CACCGTGGAAATTGGTTTATACGGTATTAACAGGTGTCATAGCAGCATTGGGTCTTA
TTGCAGGGGTTGCTTCATTGTGGCATATGTTTAAGGCCACCCCTCAGGAAAATATTG
AGGGTGTTAAATTAGCACCCGAATCCCCTTATTCTTATGATCCTCGACGTGGTCACA
AGTCTAAGGTGACCATGAAGATGGGTCGTAAGTATAATGTTATGCCTAAGTCTGGT
AAATTTAGAGGGCAGGCATTGTTGACCCACACAATTGGAGAATCGTGTAACTCCAT
AATAAAAAGGAATACTTTCTTTATTAGGGCAACATATGAACTTAATGGCGTAAGAT
CTGTGCCAATTAATGCTCGATGCCTTGGGGTTACAGGCCGATATGCCATATTCCCAT
TGCATTATAACGATGCATGGGAACGTCTCAATGAACTTGATGGCGTTAAATTTGAGT
ATTATGATGTGACAACACCTATACCCATTGAGTTCGCGTATGATGAAATTGACATAT
TGGCGTCAGATGATTCAGCAGCCCTGGCGTTTATTCGTATGCCAAAACGTTTTAGGG
TGTTTAGGGATATTAGAAATCATTTTATCCCTAATGATAAAATTTGTGTGGGGAAGA
CAAAGGCATGTCTTTTTGAGTTGAAAATTGATTTAAATAAAAATTTAACTACGATAG
ATAGGCATAACTTTGAAGCTGAACGCTCTGAGAGCTGTAAAATCCCAGCCTCAGAG
CATTTTAATGAATTTACTGTCTCGTCGGCCTATAGGTATGCGTATGGCGGTGCGGGT
GTATGTGGTAGTGTGCTGTTTGACTGGATGGGCAATATAATTGGTATGCATGTGGCT
GGTTTGGGTGATGGATCAGTGGGTATGAGTGAATGGTTAACTGAGACCGATATGAA
TGATCTTACTAATCTTATGTCGGAGTTAGAGGATCCTGTGGGTGAAGTAGAGTTTAA
TCCAGAAATTTTACCAGAAAAACCTGATGAACTTGTGATGAATCCGACATGCCAGG
TTCTGGATATTGGTATGATTCACCCATCTTTCAGAAATTTTGTGGATAAAGATTTCA
AGTTAATTAGGAGTCCGGTTCATAAATTGGCTGAAATAGAACACTTTCCAGATCTAG
TAGAACCTGCTATACTGGATCAGCGTGATAATAGGTATCAATATAGTTCGTCACCTC
TATTTGCGGGCCTTAAGCATCATGGGTGTCCAATGAGACCGTTACCGTTGGAGATGA
TCGAAGAGGCTGTCGGACACTACACGCACAAAGTACTTACTTTGTGTAGACCTGTGC
GCCCAGAACCGGCTATCCTTTCGGTGGAGGAGGCTTGTATGGGCGTGGAGGGAGTT
AGACTGATAGCCCCTCTTGATTGGGGATCTTCTGCGGGATGGCCTTGGAGGATATCT
CGTGATGTTATTAATCCGGCAACAAAATCTTCGTGGGTAAAATTGACTAACGAAAA
TACTAAATTTATTGGCCTTCATCCAGATTTGGAGGAGGTGATGAACATTAAACATGA
ACAACGCGTGGCTGGAATCGTTCCACTGACGGTGTTTGCTGACATATTGAAGGACG
AATTGGTGCCTCATGCAAAAGCAATGCGTGAGGGCGCGACGAGGATTGTCTCGATG
AGCCCCATCGACTTCACTATTCAGCAGCGACAATACACCATGGATTTCGTCGCGTCA
TTCATGGAGAATAGATTGGATCTGGAACATGCGATAGGTATCAATCCGGACTCAAG
GGAATGGACAGACCTCTATGTTAAATTAGCAAAAGGAGGTAAGACCAAATTCATAG
CAGGGGATTATTCTAAATTTGGGGATAAACTTCCGACCGAGATTGGCGTGCGGATG
TTTGGTGTGATTGGTGCGTGGTATGCCCGGTATGCACCCCCGGAGCAAGTATGTATT
ATACAGAGGGTGCTTTCTATTATGGCCTTAGAGGTCTTCAATGCAAAACACATTGCG
GACCGTTGGGTTTATCAGTGTGTGAATGGATTGCCAAGTGGTAACACCATGACCGTA
GTTTTAAATTCAATGGTCAATTCCATTTTTATTCGAATCGCGTGGATGGCCATTATGT
CCAACACAGAATTTAGTGGTTTGGATAATTTTGAAAAGTATACTAGTCTATATTCGT
ATGGTGATGATGTTATCATTGGCTTGGATGATTGTGTATGCGACTTGTTCAATACGC
TAACAATTAGGGATTTCTTTGCCAGGTACGATTTAAAATTTACTGATGCAGATAAGG

AAGGTATTCCAGTTAAATATAGGACATTAGATGATATGACCTTCCTTAAACGTAATT
GGCTTAGGACGCACGATAGGAAATTAATTAGTTCAAGAGTATATTTTCTTGCTCCCT
TGGCCGAAGATTCAATGTTTGGCATTCTACATTGGATTAAGAAGGGGCATGAACCA
ATAGAGTTTGCCAAGGAAATGTCTGAAGAGAGTGCTCGCATGGCTTTCACCCATGG
CGAGGAATATTATAATAAACATTGTGATAAAATTAGACGTATTTGGCAAAAAATGG
GGCATTATATTTGTTTGCCCAATTGGCATGATTTAGCGTATAGAACATATTTGAGTG
ATCAACCGTTGCTGCGCTTGACGCGTAAGCTTGAAGGCTTGCCTGAAGCTTATGGCG
GACCTGCGGGCCACGCAGGTTAGCGCGGCAGGAGACCATCCTAGTTTTGAATCGAT
AGGTGTACTAGGGTGGGCCCGGGCTGGTGAAGCCCCTTATGGAGGGATTTTTATCCT
CTTTTGCATGTAGGGGTTTTTCCCCTCCATAAGGTTCCGGGTGTGGCAAACACCTAC
AAAAAAAAAAAAAAAAAAAAAAAAAAAAAAAAAAAAAAAA

Table S5- Complete VDV-3 sequence- 4202 nucleotides

CTGTGTCAGAGCGAATCTGAGTGATTTGTTTTGATTTCCATAGTGATAGTTGATCTTA
AGCAAATATGGATTTTGCAAATAGAAAAGCTACTTATAGGAATGGCGGTCGTAGCA
TGCCCGTTCCCCCCGTAAATTGTGCGTGGGGTAAGTGGAGCACGCTGCTTACCGTCG
TGCTTGTTGCGGCTACTGTAATTTATATGAATTGTATAGAGTTACAATATACGTTCG
ATTTACGAGAGATGAGGTTTGACCACATAATATGTACTTTGATCTGGACAATGGCTC
TAATATTTCTAGTGGTTAATGGAGTACTTGGGCTCGAATACCGGATTAAGAATATTA
TGTATAATATGTTCCTGCTGGATGCCCCTAAGACTACGATTTATGAAGATACAGCTA
TGTTGGAGAGTATGAAGGTTAATCCTTTGGTTACAGCTCATCCTGATACTTTCCCAA
GTTGTCAGTTAATGGTGCTTACGTGCAATGATCAGGGTGTATGGTGTAGGGCGGGTA
ATGGCTTTAGAATTTCTAAGACCATAGTTGTCCCAACACATGTTATTGATACATCCG
CTTTTACTGCAAAGTATGTGCGCTTGGCTGTACCTGAGGGGAAAAGATTAGGAAAG
ATGGTAGTAGTTGAGACGCCCATAAAGGACATATTGACCATTGGGGATGATATATC
CCTTATCAACATCTCAGAGATTGAATTGAATCGTTTAGGTGTGACAGCCGCTCGTAT
CAAGACTTTGGATACTAAGCAGACATCAGTTATGGCAATGATTTACTCCTATGCTCC
TTTGTTTAAGGCTAGCGTGGGTAAAATTAGTCCATTTGATGGTTTGGCGCAACAAAT
TGTATTTTCTGGATCGACGATTCAAGGATTCAGTGGAGCGCTTTACGTTGCGGGTAA
GAATGATGTGTATGGGATGCACACGTCAGGCGGTGCTGTGAATTTTGGCATATCGG
CTAGTTATTTGCAGCTGTTGGTACTCGGTACTGAGTCGAATGACACTATCATCAAGA
GTCAGGATAACCCCCTTAGTAGGTTGATAAAAAGATTCATGGAATTGGAAAATGGG
TCGAGTGTGACGCCAGAATCGAGGGGTGGTTATTCATCTGACAGGACGCTTGAGGA
ATTCCTTTCGGATTGGGATAACAAGAAGAAAGGAAAAATTGGGCGCAATTGGAATG
ACTCATTGGGTGCTTATGTGTATTCAATTGAAGGTAGATTCTATAATATTACTGAAG
ATATCGACAATTATGCACAGATTCGGTTTCCACATCTTTTTGAAGATGCCGATTTTG
AGTATGACACCCATGATGAGAATGGTGATAAGCGAGTTAGGCATAATAAGTTTTCG
AAATCAAAACGATATGCAAACCCAATTGAAGATGAAATTCCAGTTGAAGATATTTA
TGAAGAAGAAGAAGAATATCGCCTGGAAGAACGTAGAAGGAAGAAGGTTGAGTTT
GATGAATTCGTTAGAAAACATGAATTGGCCGGTAGTGCTTGGTGGGCTGATGAATC
AGCTACTAAGCGAGTAAAACAATCAGAGAACGTTGGTATTGGTGTTCTGTTTAGCTC
TTTGACGAAGCTCACAGCCTTGGTCGAAGACTTGAAACAAAAATGTTTGGCTGCTG
AAGTTAAGAGCGATCAATGTCATAAGGAACACCAGCAGTTGATGAAGCGGCTCTTA
ATACAAGAGGCGAATGCCAATCCAAATGGTGGATATAAGGATGATGTGTTAATGGA
GCCAATTTTCCACGGGGATTCTGAAGCTGAATTGTTGGCTGACTATAACCAAATGGT
CGCACCCATGTTGGTTGAGGATGAGTTGGATTTGTTGCGGGAAACCGTTAATAAATC
CGAAGTCAGCTACGAGGAAGTGCAGAGTATTAGATCAATCTGTAATAGACTGACTC
AACTGGGAAAGTTTTCAAATCAATTGTTGAGTACTATTGACAAGATTCGCGCTGATA
AGAAGAAACGGGAAGCTGAAGAAAGAGCTCGCCAGATTGAGATGGAATTGAAGAT

GAGGAAAAAAGAAGAACTCAAGAAGCGTGCTATTGAAATGGAAGAATCCCGGGCC
AGACAGGAGTCTGAGGCAAAGGAGAAGGAAGTGAAGCGAAGCGCAATGTGTTTAC
GTGTGCGCGAATTGTTCGAGAGTTTAACGGAAGAGGAAATTAAAGAATTCTCTAGG
CTGGTGGTCGAGAGAAATACCAAGAGGGCTCTAGCTCGTAAATCCGAAAAGGAGA
AGATGAGACAAGAAGTCATCTCGGCTATCGACCGACCAGAGGAGAAGTCCGACAA
GAAGATTGTTTTAGAAGGTACTAAGTTAAAGGACACTAAATCTTCTGTAAAGGAAG
ATATTAGACCGCCTCCCAAAGGAATGATAAGATGTCCATCTTGCATTATAAGGAAG
GCAAAGGATAGGCCTTGCTTTAGCAACAAACCAGATTTCTTTAAACACATGGAGGA
GGTTCATCTTACACGTTTGGAGGCAGCTCCTTCGGGAAACTAGGTGAGCCCGCCGGT
GGGCGGGCTTGTCCTGACGATTGTGACTGCCTCCAGATGTTTGGTATGTTTGAGAAT
CCAAGTGTAAGGAATGATTGTTGTGGTAAAATGCATTGTATGAGAGATTTGTGGATT
CCTTTGCGGAATACCCGTAAAGCTGTTAAGAATAAATTGGCTGGTTCGTTCTATGAT
GCTGAAACTCTTAGTAGAGTATTTTCGGCAGAGGGATTCGAACACCAATTTCAATAT
CCAATGCCTAATAGCGTCGATGCTCCCCGTTATTTGGGAGCTTCACTGTGTTACCAC
GCCCGGTTAGCTTTGAGATCTCGTTCATTGTTGAAACCTCCAACCAATAAGGAAATG
GATTTGATATTGGACGATGTCGAGCGCGTTTATGGTGCGACACGCGTTTTGTGGCAT
GGTGTTGAGGAGGTAGATGATGATTTAATGGCTGAATTTATGTTGAAGTTGGATAG
GGACGCCAGTCCGGGTTATCCTTGGAAAGATGAGGCGCCCACCAGTGAGGTTTTAT
TTGGTCTTGATGCAGCCCATCTTCCACACAATTACACCAATGTCATGCGCGTTAAAG
AATCTGTAAATGCCAGATTACGTGTCTTGAGGAAGGGAGTGGTTGAAGCCGACAAC
ATCAACGTCTTCATAAAACAAGAGCCACATAAGATGAAGAAGGTTGATGATGGAGC
GTGGAGACTGATACAAGGAGTGGGTTTGACTGATAATTTAGTTGATCGGCTTATTTT
TACACACTGGTTTGAGGCGTTAATTGAGAAACACCGCAGTGTACCTAGTAAACCAG
GTTGGGCACCCATCAAGGGAGGTTTTCGTTGGTTGGCTAATCAGTTTAGACGACCGA
TGATGGCTGATAAGTCAGGATGGGATTGGACAGTACAAGAATGGCATGTGGACTTC
CTGAAACGGTTCATACCCCGAATGATAGTTGGGGTCCCAGAAGTTTTGGTGAATTCG
AGAATTGATGCCTTGCTTGAGGAGTGTAAATGGCAGTTAGCAGATACAGTCATAGT
GCAGAATGCCATGGGTGTGCAGAAGAGTGGTTGTTTTGGCACCATTGCTTTCAACAG
CCTATGGCAGTTTGCAGTCCATGCTCTGGTTTGTCGGAGATTAGGCTGGTCTTTGGA
GAGACTTGGGGGGTTTGCCTGTTTGGGAGATGATACCCTTCAGGAATGTGTCCCTAA
TGTACCGGCCTATGAGAGTGAAGTTGCAAAGACTGGTGCTATTGTCAAAACCTCGG
AGATTTCTGATTTGGTTAATCCAATAAGTGAATTCGCCGGGTTTAAATTTGACAAGG
AAACCTGTCTACCGGCATATGGTGCTAAACATGCTTACATTTTATATCATGTGAGTG
AAAGTGAAGCACCAGAGACGCTTGCTAGCTATCTACTGAACTATGGCATGCATGAG
GAAATTTCAGGTTTCCTCAGGCGTGAGCTTATCAGGCTGGGTTATCCAGAATTTGCG
TTGAGTAAAGACATGCTCAAGCATTTCTGGACTGGCTTGGAATAGGCTCTGGGTGTT
GCAGCACCCTGGTACCACTCTTTCCCTGGGAGTGGAACCTTGCCACTAGGATTGGTC
ACCAAGTGGTATGTCCGTCGTGAATCGGATCCCGGGCCCGTCGACTGCAGAGGCCT
GCATGCAAGCTTTCCCTATAGTA

Table S6- Blastx analysis of *A. mellifera* contigs

Contig ID	Description	Length	#Hits	e-Value	sim mean
c6358_g3_i1	polyprotein [Varroa destructor virus-1]	7664	5	0.00E+00	97.40%
c6350_g2_i3	polyprotein [Deformed wing virus]	2069	5	0.00E+00	97%
c6350_g2_i1	polyprotein [Varroa destructor virus-1]	1997	5	0.00E+00	97%
c6350_g2_i2	polyprotein [Deformed wing virus]	1911	5	0.00E+00	97%
c6355_g1_i1	structural polyprotein [Black queen cell virus]	3062	5	0.00E+00	96.80%
c6355_g1_i2	structural polyprotein [Black queen cell virus]	1970	5	0.00E+00	96.60%
c6355_g2_i2	non-structural polyprotein [Black queen cell virus]	1758	5	0.00E+00	96.40%
c5497_g1_i1	BCL2 adenovirus E1B 19 kDa -interacting 3-like	581	5	1.70E-95	96%
c8148_g1_i1	tobamovirus multiplication 3-like isoform X1	395	5	1.20E-57	96%
c9570_g1_i1	xenotropic and polytropic murine leukemia virus receptor ids-4,	201	5	1.30E-33	95.20%
c6355_g2_i1	non-structural polyprotein [Black queen cell virus]	4528	5	0.00E+00	94.40%
c6359_g3_i1	polyprotein [Sacbrood virus]	7622	5	0.00E+00	93.80%
c7894_g1_i1	tobamovirus multiplication 3-like isoform X1	245	5	4.00E-44	93%
c457_g1_i1	feline leukemia virus subgroup C receptor-related 2-like	362	5	1.10E-73	92.20%
c722_g1_i1	capsid protein [Lake Sinai virus 1]	2826	5	0.00E+00	89.60%
c2876_g1_i1	capsid protein [Lake Sinai virus 1]	3040	5	0.00E+00	88.80%
c7183_g1_i1	tobamovirus multiplication 3	291	5	5.50E-40	86.20%
c16552_g1_i1	xenotropic and polytropic murine leukemia virus receptor ids-4,	242	5	1.50E-17	84.60%
c8697_g1_i1	tobamovirus multiplication 2A	268	5	3.10E-15	80.80%
c6357_g2_i1	coat protein [Grapevine Bulgarian latent virus]	2483	5	0.00E+00	75%
c6357_g1_i1	polyprotein segment 2 [Grapevine Bulgarian latent virus]	2414	5	1.10E-150	66.20%
c6346_g1_i1	L [Farmington virus]	14606	5	0.00E+00	45.80%

Table S7- Blastx analysis of *V. destructor* contigs

Contig ID	Description	Length	#Hits	e-Value	sim mean
c11085_g1_i1	polyprotein, partial [Deformed wing virus]	409	5	2.80E-91	100%
c10331_g1_i1	capsid protein, partial [Deformed wing virus]	277	5	1.10E-58	100%
c11078_g1_i1	polyprotein [Deformed wing virus]	252	5	6.50E-55	100%
c10951_g1_i1	polyprotein [Deformed wing virus]	322	5	3.50E-51	99.60%
c24131_g1_i1	structural polyprotein [Sacbrood virus]	413	5	6.80E-92	99.40%
c24947_g1_i1	non-structural polyprotein [Black queen cell virus]	355	5	1.20E-71	99.40%
c5466_g1_i1	polyprotein [Sacbrood virus]	781	5	8.70E-171	99.20%
c11085_g2_i1	polyprotein [Deformed wing virus]	1712	5	0.00E+00	99%
c19779_g1_i1	structural polyprotein, partial [Black queen cell virus]	208	5	1.70E-37	98.80%
c4948_g1_i1	polyprotein [Deformed wing virus]	211	5	9.80E-36	98.40%
c18557_g1_i1	PREDICTED: tobamovirus multiplication protein3-like [Nicotiana sylvestris]	208	5	1.20E-38	98%

c11088_g2_i3	structural polyprotein [Black queen cell virus]	835	5	0.00E+00	97.80%
c11057_g2_i1	polyprotein, partial [Deformed wing virus]	284	5	1.10E-58	97.80%
c11093_g1_i1	polyprotein [Varroa destructor virus-1]	9164	5	0.00E+00	97.20%
c9198_g1_i1	polyprotein [Sacbrood virus]	821	5	1.10E-180	97%
c14161_g1_i1	polyprotein [Sacbrood virus]	594	5	5.70E-112	97%
c11088_g2_i2	structural polyprotein [Black queen cell virus]	4932	5	0.00E+00	96.80%
c704_g1_i1	non-structural polyprotein [Black queen cell virus]	578	5	9.50E-123	96.80%
c11088_g2_i1	structural polyprotein [Black queen cell virus]	398	5	1.70E-82	96.60%
c11087_g2_i1	polyprotein [Sacbrood virus]	2431	5	0.00E+00	96.20%
c28455_g1_i1	non-structural polyprotein [Black queen cell virus]	439	5	2.20E-86	96%
c11087_g1_i1	polyprotein [Sacbrood virus]	608	5	3.90E-117	94%
c11044_g1_i1	polyprotein [Deformed wing virus]	559	5	9.10E-111	94%
c4210_g1_i1	non-structural polyprotein [Black queen cell virus]	1072	5	0.00E+00	93.60%
c11057_g3_i1	polyprotein, partial [Deformed wing virus]	223	5	1.90E-37	93.60%
c11074_g1_i1	replicase polyprotein [Acute bee paralysis virus]	9590	5	0.00E+00	92.40%
c10756_g1_i1	P15 [Varroa destructor Macula-like virus]	252	5	9.60E-42	90.20%
c10756_g1_i2	P15 [Varroa destructor Macula-like virus]	220	5	6.00E-37	89%
c10988_g1_i1	polyprotein [Deformed wing virus]	355	5	8.40E-60	88%
c15340_g1_i1	RNA-dependent RNA polymerase [Varroa destructor Macula-like virus]	269	2	2.90E-50	88%
c20855_g1_i1	structural polyprotein, partial [Israeli acute paralysis virus]	219	5	7.00E-30	83%
c22187_g1_i1	non-structural polyprotein [Black queen cell virus]	710	5	4.70E-110	81.20%
c10810_g1_i1	polyprotein [Maize rayado fino virus]	421	5	3.10E-58	79.80%
c30069_g1_i1	RNA-dependent RNA polymerase [Varroa destructor Macula-like virus]	271	5	2.70E-51	79.20%
c11057_g1_i1	polyprotein, partial [Deformed wing virus]	224	5	2.90E-31	78.20%
c19782_g1_i1	polymerase acidic subunit [Aransas Bay virus]	319	5	5.10E-29	78%
c1503_g1_i1	putative replication-associated protein 2 [Florida woods cockroach-associated cyclovirus]	210	5	6.50E-20	77.80%
c23590_g1_i1	polyprotein [Maize rayado fino virus]	378	5	3.50E-49	77%
c1508_g2_i1	replication-associated protein [Anguilla anguilla circovirus]	325	5	5.10E-33	77%
c10878_g1_i1	RNA-dependent RNA polymerase [Varroa destructor Macula-like virus]	638	5	4.50E-106	74.80%
c4341_g1_i1	PB1, partial [Jos virus]	305	5	6.30E-29	72%
c16161_g1_i1	replicase protein [Erysimum latent virus]	221	5	1.40E-17	71.20%
c6211_g1_i1	polymerase basic subunit 1 [Upolu virus]	304	5	7.20E-27	71%
c359_g1_i1	PREDICTED: influenza virus NS1A-binding protein homolog A-like [Metaseiulus occidentalis]	243	5	3.90E-25	70.20%
c4939_g1_i1	RNA-dependent RNA polymerase [maize rayado fino virus]	411	5	7.00E-38	69.20%

c31128_g1_i1	GP64 [Epiphyas postvittana nucleopolyhedrovirus]	248	5	1.60E-20	68.20%
c8474_g1_i1	RNA-dependent RNA polymerase [Turnip yellow mosaic virus]	770	5	2.90E-75	67%
c48_g2_i1	gp67 [Choristoneura fumiferana multiple nucleopolyhedrovirus]	250	5	1.30E-16	65.80%
c10913_g1_i1	polyprotein [Brevicoryne brassicae picorna-like virus]	235	5	1.60E-14	65.20%
c8205_g1_i2	non-structural protein [Spodoptera exigua iflavirus 1]	520	5	2.20E-17	63.80%
c20706_g1_i1	PB2 polymerase subunit [Thogoto virus]	216	5	2.10E-08	63.60%
c10905_g4_i1	replication-associated protein [Dragonfly cyclovirus 6]	525	5	1.60E-49	63%
c9968_g1_i1	polyprotein [Plantago mottle virus]	245	5	6.30E-13	62.80%
c8514_g1_i1	replication-association protein [Cyclovirus TN25]	931	5	1.10E-46	61.20%
c1508_g1_i1	replication-associated protein [Dragonfly cyclovirus 6]	542	5	1.90E-26	61.20%
c27530_g1_i1	rep protein, partial [Circovirus-like NI/2007-3]	224	5	3.00E-09	61.20%
c21581_g1_i1	nucleoprotein [Thogoto virus]	401	5	7.70E-27	60.40%
c7702_g1_i1	replication-association protein [Cyclovirus NG8]	328	5	5.30E-15	60.40%
c8205_g1_i1	non-structural protein [Spodoptera exigua iflavirus 1]	457	5	5.30E-09	59%
c10905_g3_i2	replication-associated protein [Dragonfly cyclovirus 6]	917	5	6.70E-37	58.40%
c8514_g2_i1	replication-association protein [Cyclovirus TN25]	740	5	2.70E-33	58%
c11347_g1_i1	polyprotein [Heliconius erato iflavirus]	272	5	3.50E-11	57.80%
c27684_g1_i1	polyprotein [Heliconius erato iflavirus]	344	5	7.70E-18	57.40%
c11061_g1_i1	polyprotein [Brevicoryne brassicae picorna-like virus]	782	5	3.90E-34	56.40%
c10876_g2_i1	non-structural protein [Spodoptera exigua iflavirus 1]	796	5	3.90E-12	56.20%
c7507_g1_i1	polyprotein [Formica exsecta virus 2]	238	5	6.30E-08	56.20%
c11086_g2_i2	polyprotein [Halyomorpha halys virus]	437	1	8.80E-08	56%
c11092_g1_i2	putative polyprotein [Tomato blistering mosaic virus]	5058	5	0.00E+00	55.40%
c11083_g2_i1	polyprotein [Sacbrood virus]	715	5	3.10E-25	55%
c11553_g1_i1	polyprotein [Antheraea pernyi iflavirus]	422	5	1.10E-16	54%
c16528_g1_i1	glycoprotein [Jos virus]	315	5	7.40E-14	53.80%
c2745_g1_i1	ENV_DHVI1RecName: Full=Envelope glycoprotein; Flags: Precursor [Dhori virus (strain Indian/1313/61)]	1185	5	5.60E-64	53%
c18230_g1_i1	nucleoprotein [Aransas Bay virus]	380	5	1.10E-19	51.80%
c11092_g1_i1	polyprotein [Plantago mottle virus]	6436	5	0.00E+00	51.20%
c11050_g1_i1	polyprotein [Sacbrood virus]	433	5	1.50E-08	51%
c11086_g2_i4	polyprotein [Halyomorpha halys virus]	442	5	3.80E-09	50.20%
c11083_g3_i3	polyprotein [Slow bee paralysis virus]	690	5	1.30E-13	49.80%
c11029_g1_i2	polyprotein, partial [Bat feces associated picorna-like virus SC2797]	374	3	2.70E-09	49.67%

c11089_g1_i1	hypothetical protein, partial [Ixodes scapularis associated virus 2]	4169	5	6.20E-51	49%
c11029_g1_i1	polyprotein, partial [Bat feces associated picorna-like virus SC2797]	417	5	5.70E-10	47.60%
c11009_g1_i1	polyprotein [Formica exsecta virus 2]	478	3	8.30E-10	47.33%
c11083_g4_i1	polyprotein [Slow bee paralysis virus]	1269	5	2.70E-18	46.40%
c11094_g1_i1	polyprotein [Brevicoryne brassicae picorna-like virus]	4574	5	2.70E-86	46%
c7844_g1_i1	polyprotein [Slow bee paralysis virus]	714	2	1.60E-06	43.50%
c6947_g1_i1	polyprotein [Slow bee paralysis virus]	499	1	5.80E-06	43%
c11080_g1_i1	capsid protein [Mosinovirus]	1573	2	1.20E-31	41%

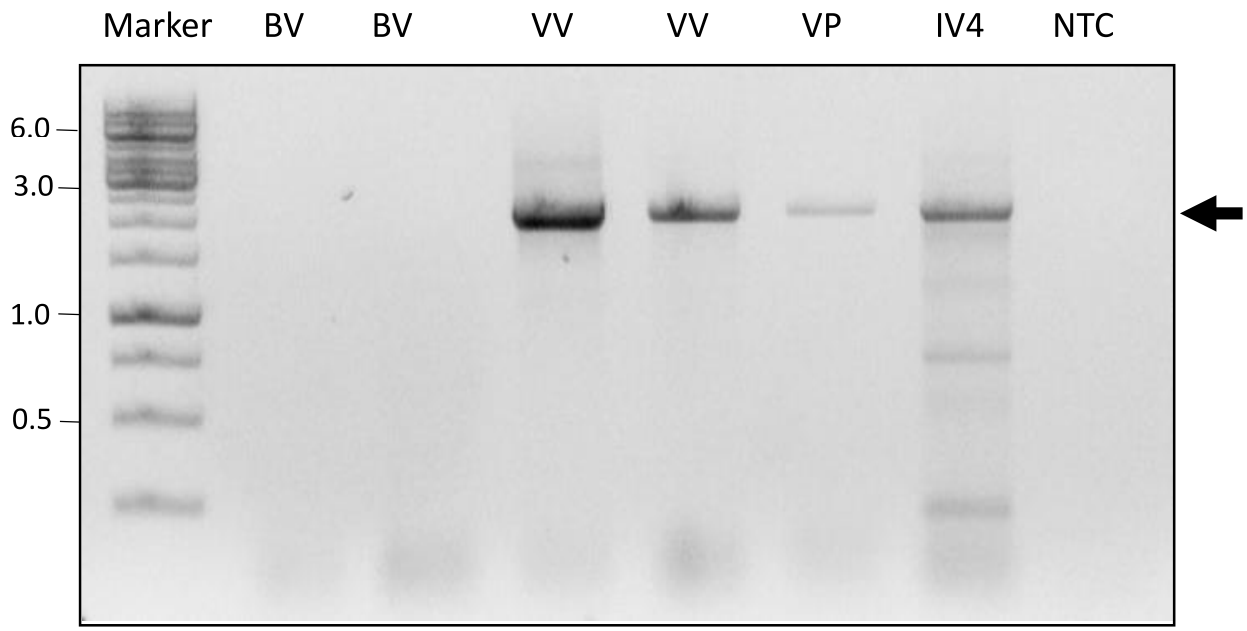

Figure S1. VDV-2 is present in the *Varroa* virome but not in the honey bee virome. RT-PCR run of the honey bee and *Varroa* viromes (BV and VV labels, respectively). VP, sample from a pool of 30 *Varroa* mites randomly collected. IV-4, *Varroa* transcriptome sample. NTC, primers only. VDV-2 primers used were 3777F and 5879R (see Supplementary Table S1). Arrows VDV-2. Marker: GeneRuler 1 kb DNA Ladder (Thermo Scientific Inc.).

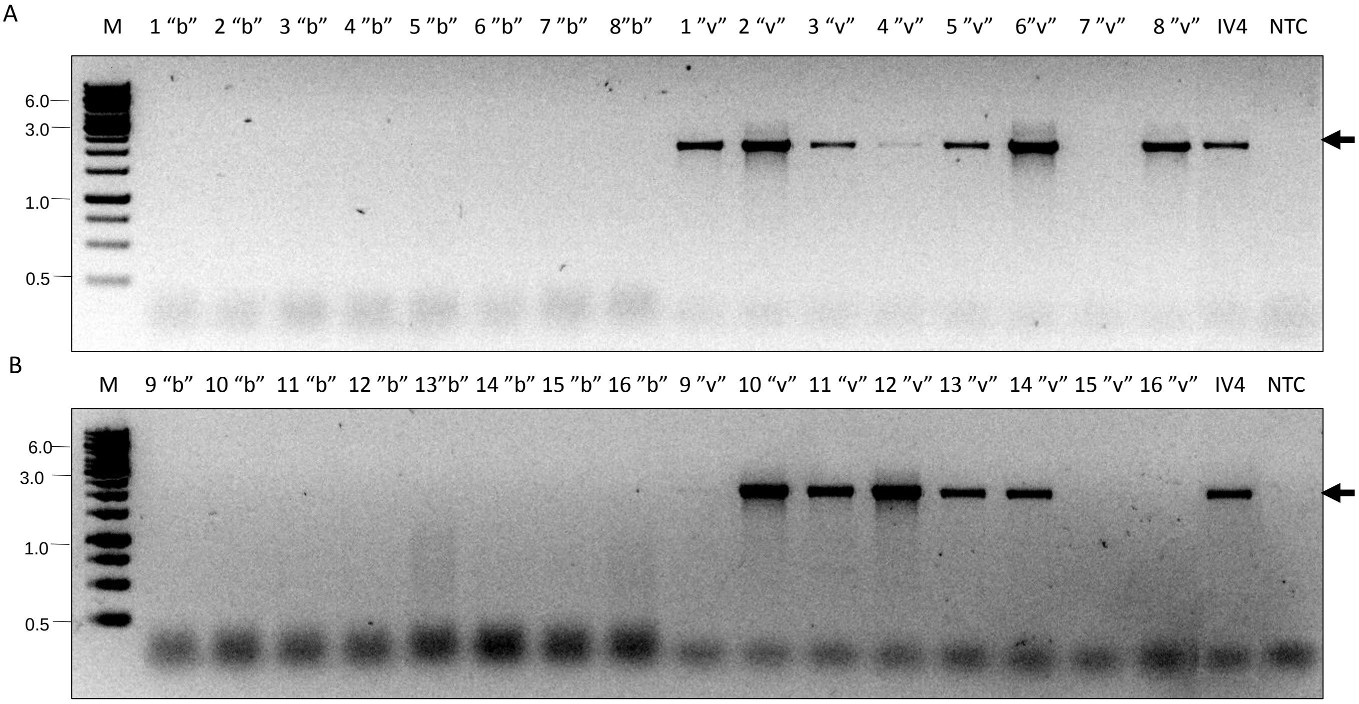

Figure S2. VDV-2 is present in *Varroa* mites but not in honey bees. RT-PCR run of individual bees (“b”) and *Varroa* mites (“v). A. Samples from hives 401 and 7 (1-4 and 5-8, respectively). B. Samples from hive 10 and 6 (9-10 and 11-14, respectively). IV-4, *Varroa* transcriptome sample. NTC, primers only. VDV-2 primers used were 3777F and 5879R (see Supplementary Table S1). Arrows VDV-2. Marker: GeneRuler 1 kb DNA Ladder (Thermo Scientific Inc.).

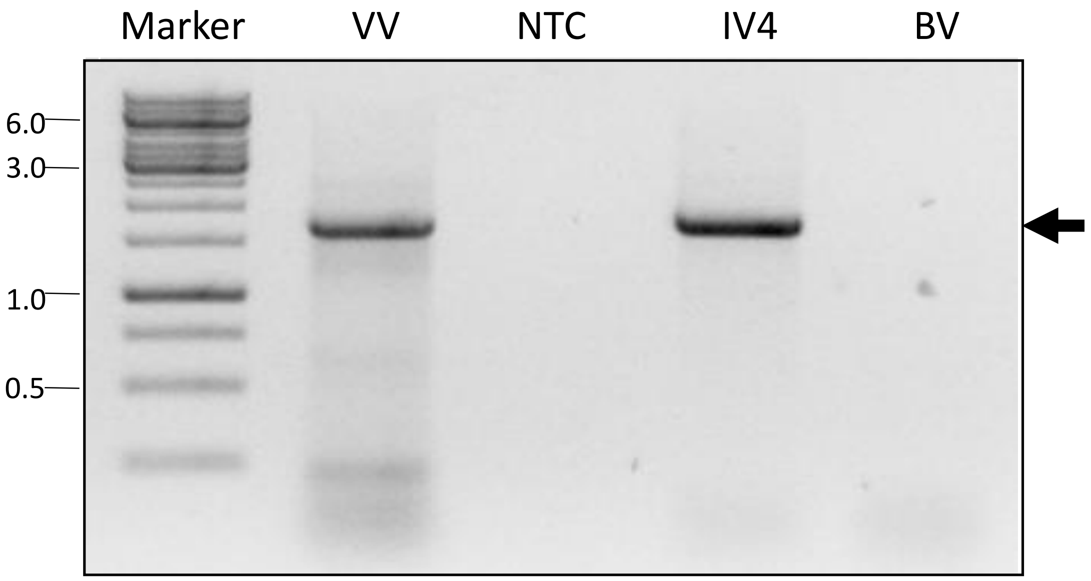

Figure S3. VDV-3 is present in the *Varroa* virome but not in the honey bee virome. RT-PCR run of the honey bee and *Varroa* viromes (BV and VV labels, respectively). IV-4,

Varroa transcriptome sample. NTC, primers only. VDV-3 primers used were 1602F and 3369R (see Supplementary Table S2). Arrows VDV-3. Marker: GeneRuler 1 kb DNA Ladder (Thermo Scientific Inc.).

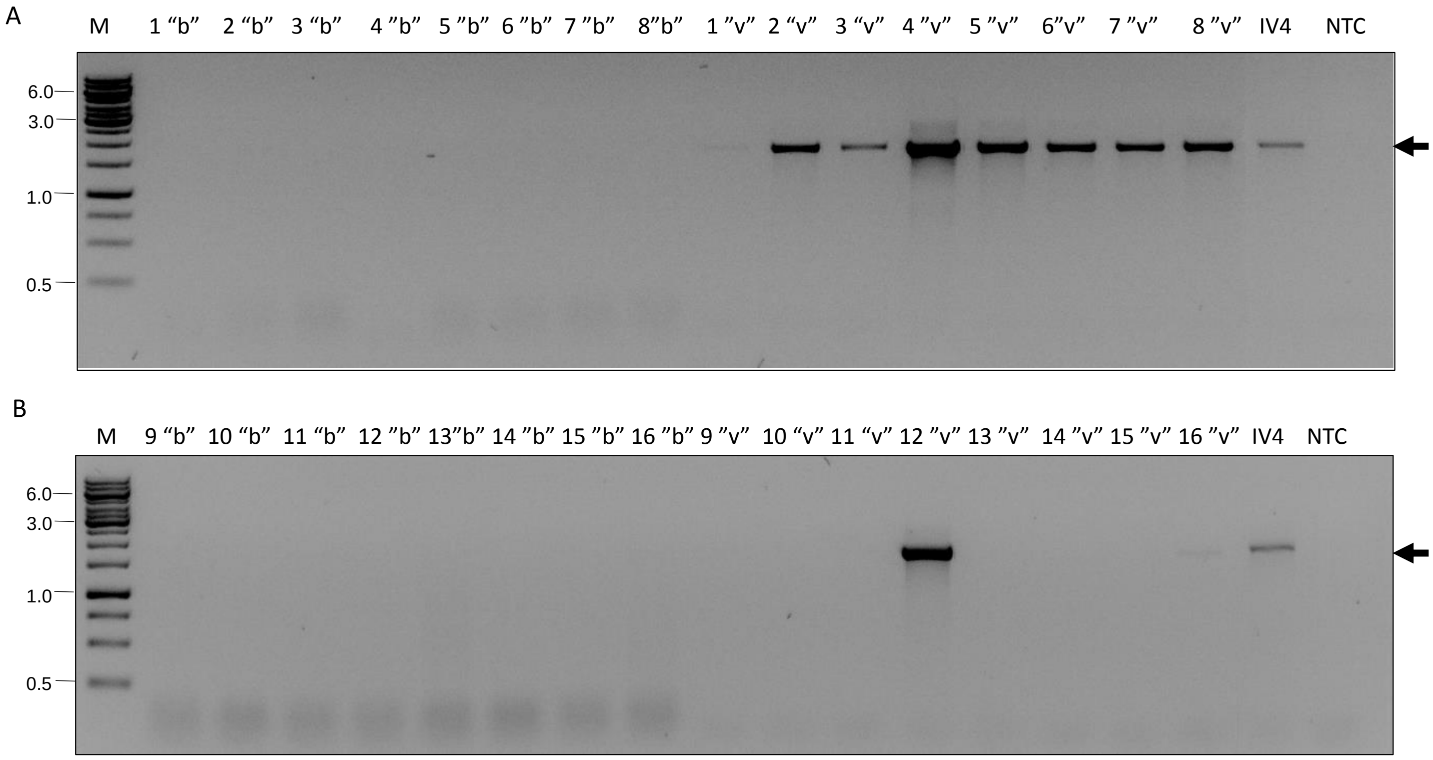

Figure S4. VDV-3 is present in *Varroa* mites but not in honey bees. RT-PCR run of individual bees (“b”) and *Varroa* mites (“v). A. Samples from hives 401 and 7 (1-4 and 5-8, respectively). B Samples from hive 10 and 6 (9-10 and 11-14, respectively). IV-4, *Varroa* transcriptome sample. NTC, primers only. VDV-3 primers used were 1602F and 3369R (see Supplementary Table S2). Arrows VDV-3. Marker: GeneRuler 1 kb DNA Ladder (Thermo Scientific Inc.).

A

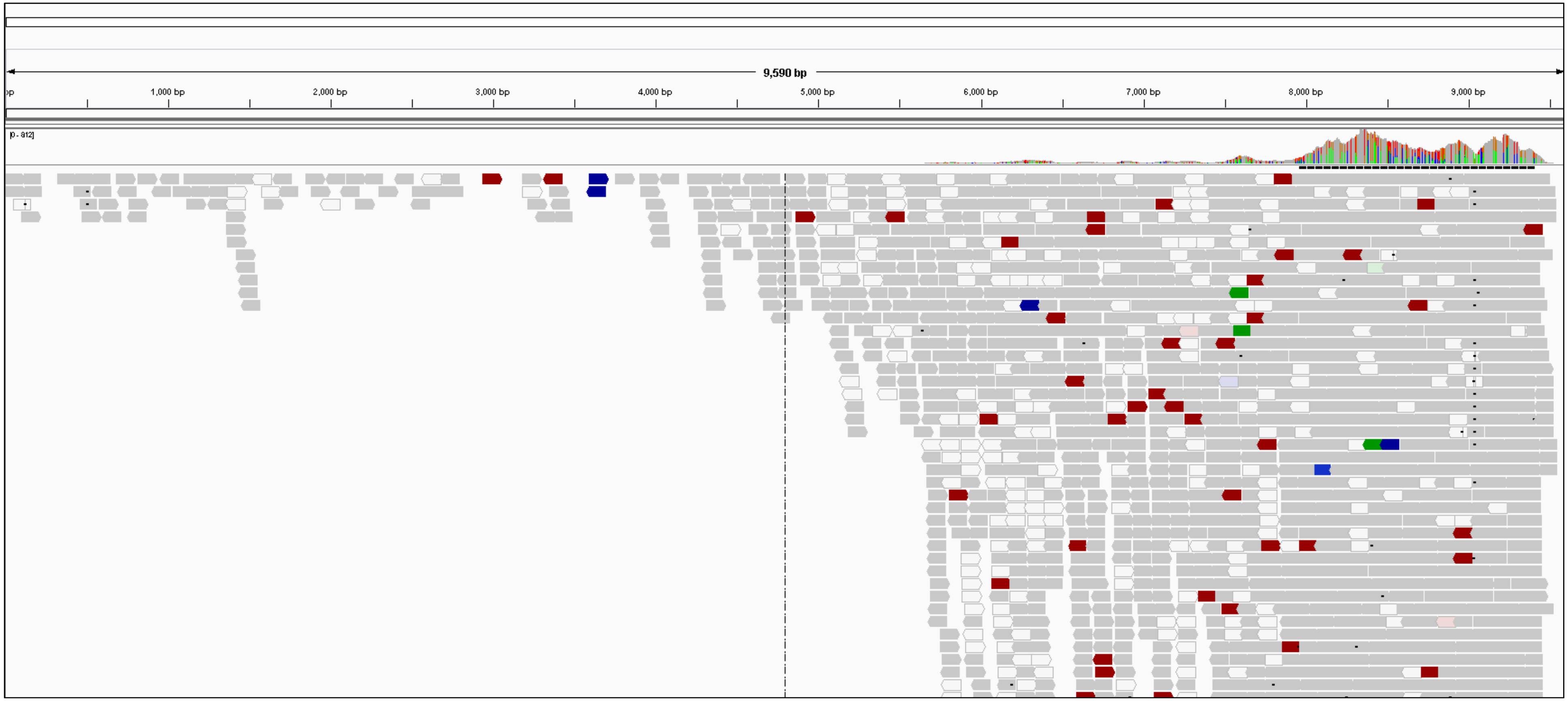

Figure S5. A. Mapping of transcriptome reads to the VDV-2 genome (A) and Virome (B). Colored bars indicate changes compared to the reference sequence as well as direction of the read sequence.

B

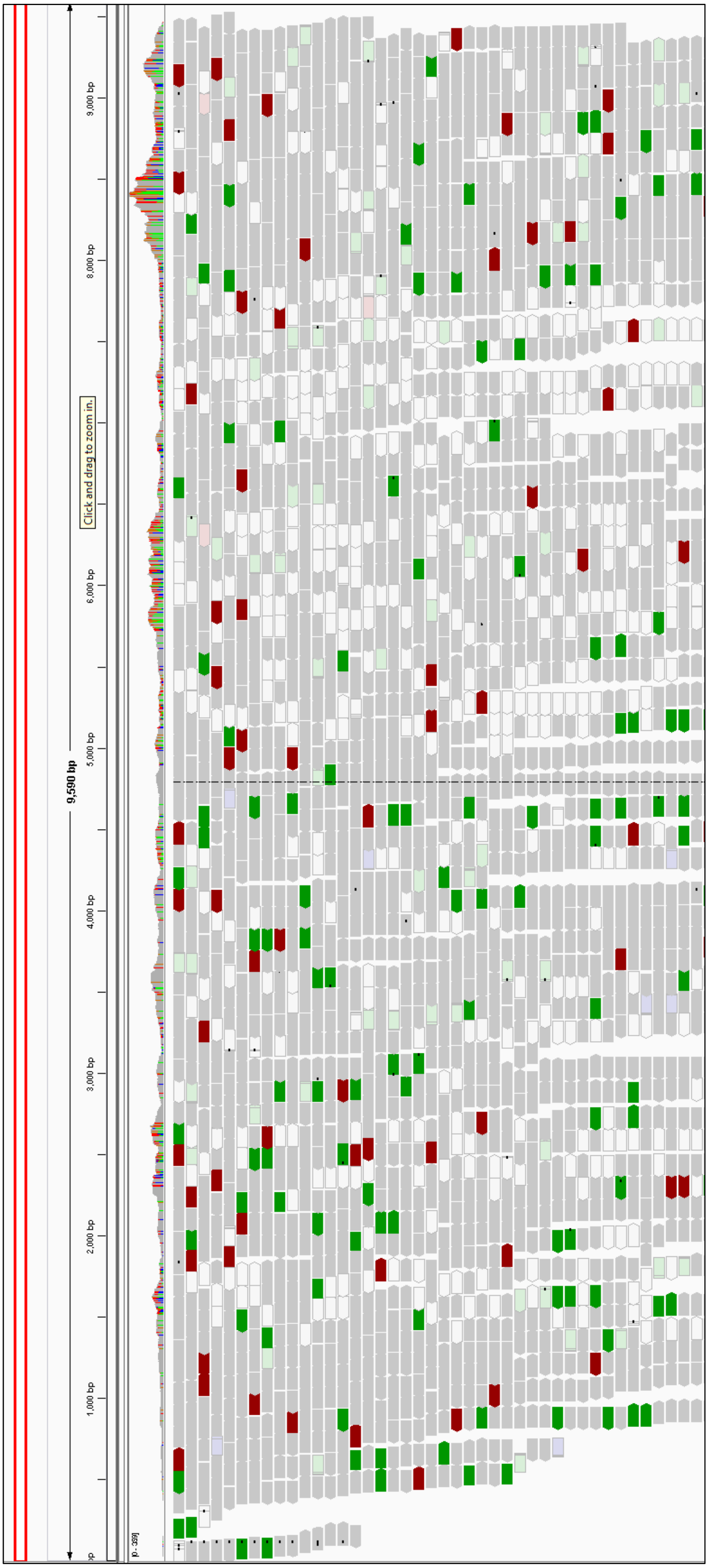

9,590 bp
1,000 bp
2,000 bp
3,000 bp
4,000 bp
5,000 bp
6,000 bp
8,000 bp
9,000 bp
Click and drag to zoom in.

A

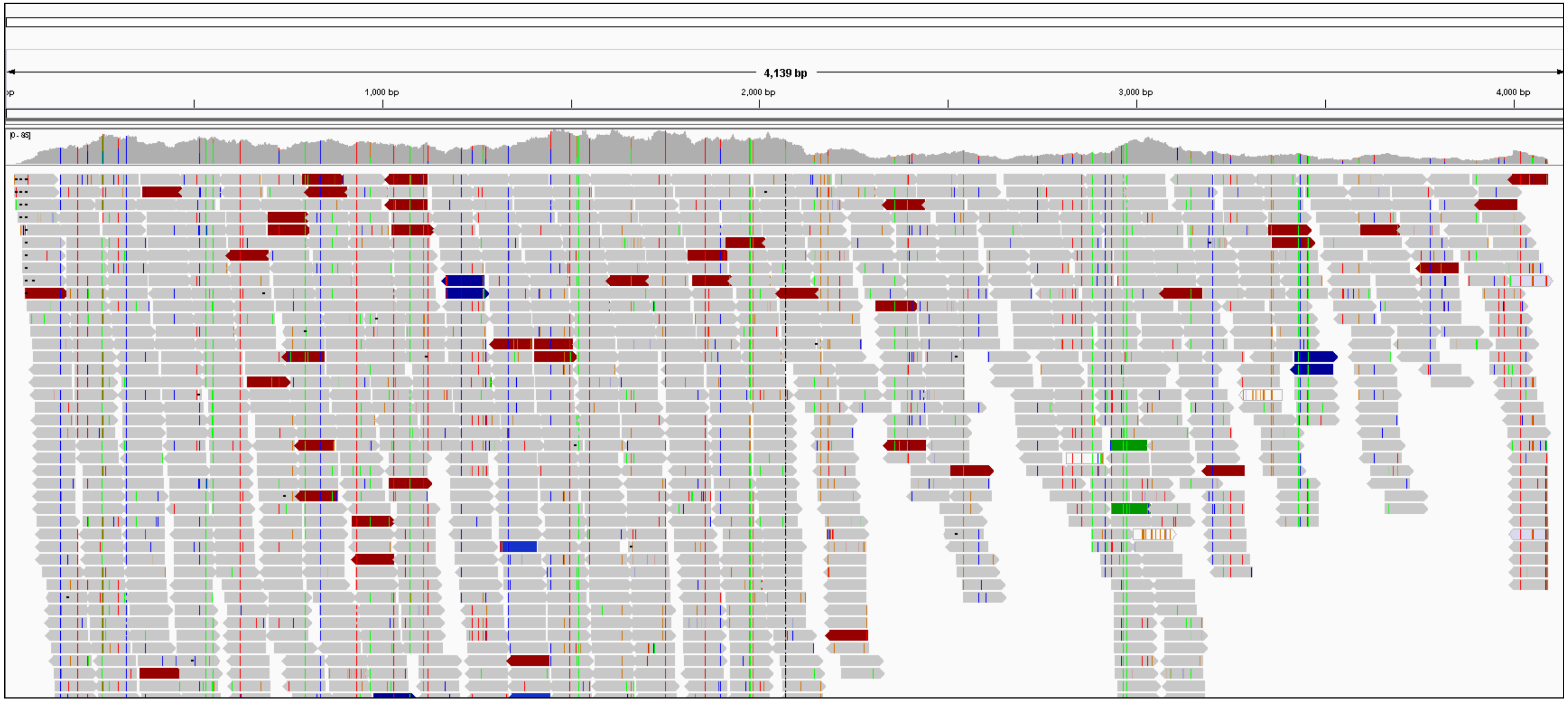

Figure S6. A. Mapping of transcriptome reads to the VDV-3 genome (A) and Virome (B). Colored bars indicate changes compared to the reference sequence as well as direction of the read sequence.

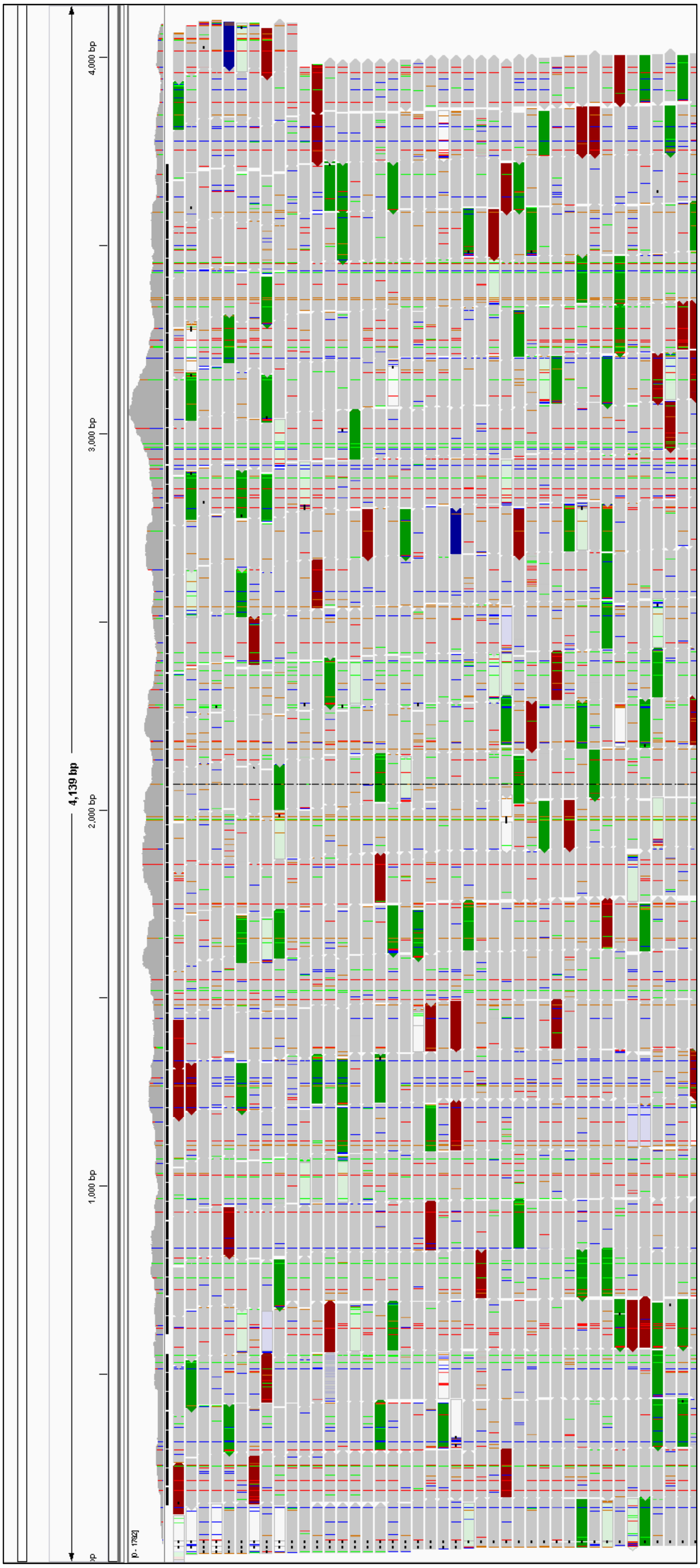

B
